# Big conductance calcium‐activated potassium channel openers control spasticity without sedation

**DOI:** 10.1111/bph.13889

**Published:** 2017-07-07

**Authors:** David Baker, Gareth Pryce, Cristina Visintin, Sofia Sisay, Alexander I Bondarenko, W S Vanessa Ho, Samuel J Jackson, Thomas E Williams, Sarah Al‐Izki, Ioanna Sevastou, Masahiro Okuyama, Wolfgang F Graier, Lesley A Stevenson, Carolyn Tanner, Ruth Ross, Roger G Pertwee, Christopher M Henstridge, Andrew J Irving, Jesse Schulman, Keith Powell, Mark D Baker, Gavin Giovannoni, David L Selwood

**Affiliations:** ^1^ Neuroimmunology Unit, Blizard Institute, Barts and the London School of Medicine and Dentistry Queen Mary University of London London UK; ^2^ Department of Neuroinflammation, UCL Institute of Neurology University College London London UK; ^3^ Department of Medicinal Chemistry, UCL Wolfson Institute for Biomedical Research University College London London UK; ^4^ Institute of Molecular Biology and Biochemistry Medical University of Graz Graz Austria; ^5^ A.A. Bogomoletz Institute of Physiology Kiev Ukraine; ^6^ Vascular Biology Research Centre. St. George's University of London London UK; ^7^ Department of Biomedical Sciences, Institute of Medical Sciences University of Aberdeen Aberdeen UK; ^8^ Neurosciences Institute, Division of Pathology and Neuroscience, Ninewells Hospital and Medical School University of Dundee Dundee UK; ^9^ Canbex Therapeutics Ltd London BioScience Innovation Centre London UK

## Abstract

**Background and Purpose:**

Our initial aim was to generate cannabinoid agents that control spasticity, occurring as a consequence of multiple sclerosis (MS), whilst avoiding the sedative side effects associated with cannabis. VSN16R was synthesized as an anandamide (endocannabinoid) analogue in an anti‐metabolite approach to identify drugs that target spasticity.

**Experimental Approach:**

Following the initial chemistry, a variety of biochemical, pharmacological and electrophysiological approaches, using isolated cells, tissue‐based assays and *in vivo* animal models, were used to demonstrate the activity, efficacy, pharmacokinetics and mechanism of action of VSN16R. Toxicological and safety studies were performed in animals and humans.

**Key Results:**

VSN16R had nanomolar activity in tissue‐based, functional assays and dose‐dependently inhibited spasticity in a mouse experimental encephalomyelitis model of MS. This effect occurred with over 1000‐fold therapeutic window, without affecting normal muscle tone. Efficacy was achieved at plasma levels that are feasible and safe in humans. VSN16R did not bind to known CB_1_/CB_2_/GPPR55 cannabinoid‐related receptors in receptor‐based assays but acted on a vascular cannabinoid target. This was identified as the major neuronal form of the big conductance, calcium‐activated potassium (BK_Ca_) channel. Drug‐induced opening of neuronal BK_Ca_ channels induced membrane hyperpolarization, limiting excessive neural‐excitability and controlling spasticity.

**Conclusions and Implications:**

We identified the neuronal form of the BK_Ca_ channel as the target for VSN16R and demonstrated that its activation alleviates neuronal excitability and spasticity in an experimental model of MS, revealing a novel mechanism to control spasticity. VSN16R is a potential, safe and selective ligand for controlling neural hyper‐excitability in spasticity.

**Abbreviations**
BK_Ca_: big conductance calcium‐activated potassium channel
C_max_: maximum concentration
EAE: experimental autoimmune encephalomyelitis
MS: multiple sclerosis
SAD: single ascending dose

## Introduction

Multiple sclerosis (MS) is the major cause of non‐traumatic disability in young adults. Disease induces neurological attacks and nerve damage leading to impaired neurotransmission and the development of a number of poorly controlled, troublesome symptoms (Compston and Coles, 2002). Spasticity is a common major debilitating consequence of MS (Barnes *et al.,* 2003) and is clinically defined as a velocity‐dependent increase in muscle tone resulting from hyper‐excitable stretch reflexes, spasms and hypersensitivity to normally innocuous sensory stimulations (Trompetto *et al.,* 2014). The intermittent or sustained involuntary muscle hyperactivity that characterizes spasticity is associated with upper motor neuron lesions that can be located anywhere along the path of the corticospinal (pyramidal) tracts from the brain to the spinal cord (Baker *et al.,* 2012; Trompetto *et al.,* 2014). Whilst the aetiology of spasticity in MS has been relatively little studied, this contrasts to experimental spasticity caused by spinal cord injury, where persistent inward sodium and calcium currents and changes in chloride ion balance in motor neurons and increases in the number of primary afferent projections to the motor nerves are believed to be the key mechanisms mediating spasticity (Li *et al.,* 2004; Toda *et al.,* 2014; Trompetto *et al.,* 2014). Clinically, these are currently treated using either nerve blocking procedures or pharmacological agents, such as depolarizing‐ion channel inhibitors, GABA_A_ and GABA_B_ receptor agonists, α_2_‐adrenoceptor agonists, dantrolene within the muscle, and more recently nabiximols (medical cannabis) to target the cannabinoid CB_1_ receptor in the periphery and CNS (Baker *et al.,* 2000; Shakespeare *et al.,* 2003; Novotna *et al.,* 2011; Baker *et al.,* 2012; Pryce *et al.,* 2014). Whilst these pharmacological treatments are active, their use is associated with dose‐limiting side effects that limit compliance and early adoption following the development of spasticity. (Shakespeare *et al.,* 2003; Novotna *et al.,* 2011; Baker *et al.,* 2012).

We have established a relapsing progressive experimental autoimmune encephalomyelitis (EAE) model of MS in ABH mice as a system for studying spasticity (Baker *et al.,* 2000; 2012). This model responds to standard spasticity therapies (Baker *et al.,* 2012; Hilliard *et al.*, 2012) and was utilized to provide the initial experimental demonstration that cannabis and the endocannabinoid system could control spasticity (Baker *et al.,* 2012; Hilliard *et al.,* 2012). The endocannabinoid signalling pathway is part of a much wider lipid signalling system with pleiotropic actions in the CNS, cardiovascular and immune systems (Howlett *et al.,* 2002). **Anandamide** is one of the key endogenous endocannabinoid mediators, and a close analogue, **methanandamide**, can inhibit spasticity in EAE (Baker *et al.,* 2000). As anandamide is rapidly degraded *in vivo* (Howlett *et al.,* 2002), by using an anti‐metabolite approach to create a more stable, drug‐like molecule with a good potential to interfere with cannabinoid signalling and avoid brain‐mediated side effects, we synthesized a series of monocyclic alkyl amides (Hoi *et al.,* 2007) based on constrained conformations of anandamide (Berglund *et al.,* 2000; Supporting Information Figure S1). We found that one compound 3‐(6‐(dimethylamino)‐6‐oxohex‐1‐enyl)‐N‐(1‐hydroxypropan‐2‐yl)benzamide, (VSN16; Hoi *et al.,* 2007) has potent activity in tissue‐based cannabinoid receptor assays yet was active in CB_1_ receptor deficient mice, pointing to a new target. We identified the neuronal form of the big conductance calcium‐activated potassium channel (BK_Ca_) as the target for VSN16 and demonstrated that its activation alleviates neuronal excitability and spasticity in an experimental model of MS.

## Methods

### Animals

Adult Biozzi male and female ABH mice were from stock bred either at the Institute of Neurology (ION) or Queen Mary University of London (QMUL) or were purchased from Harlan UK (Bicester, UK). Congenic‐ABH CB_1_ receptor (Pryce *et al.,* 2003) or G‐protein 55 receptor (GPR55)‐deficient mice (Sisay *et al.,* 2013) were from stock bred either at ION or QMUL. Outbred mice and rats were purchased from Charles Rivers (Margate, UK) or Harlan UK. Animal work was performed following an ethical review by the Local Animal Welfare and Ethical Review Bodies and the UK Government Home Office. Animal experiments were performed under the Animals (Scientific Procedures) Act 1986 and European Union Directives 86/609/EEC and EU 2010/63/EU. Animal housing and other elements related to documenting protocols of *in vivo* experiments were as reported previously (Al‐Izki *et al.,* 2012). Animal studies are reported in compliance with the ARRIVE guidelines (Kilkenny *et al.*, 2010; McGrath and Lilley, 2015).

### Humans

Healthy volunteers were enrolled in a double‐blind, placebo‐controlled Phase I safety study, (EudraCT 2013‐002765‐18) following ethical review of the project (Office of Research Ethics Northern Ireland) and informed consent was obtained that complied with the Declaration of Helsinki, GCP (CPMP/ICH/135/95) and local regulation. At the time of designing the study, reproductive toxicity had not been completed. Therefore for safety reasons and ease of recruitment, only males were studied. Individuals were screened negative for amphetamine, ecstasy, benzodiazepines, ethanol, methadone metabolites, cannabinoids, barbiturates, cocaine, urine creatinine, opiates, cotinine and tricyclic anti‐depressants. Individuals were randomized to receive either 25, 50, 100, 200, 400 or 800 mg (*n* = 6 per group) formulated VSN16R or placebo capsules (*n* = 2 per group) as part of a single ascending dose study, or multiple ascending dose study with twice a day 25, 100 and 400 mg VSN16R for 1 week (*n* = 6 per group). A randomization schedule was provided to the pharmacist, but study personnel were blinded to this. These people fasted before dosing, except one group that were fed a standardized high fat breakfast within 0.5 h prior to dosing (*n* = 6). Serial plasma samples using EDTA anti‐coagulant were collected. This study was performed by Quintiles Limited, London, UK. The sample size was based on experience from previous similar Phase 1 studies with other compounds to obtain adequate safety, tolerability and pharmacokinetics data to achieve the objectives of the study while limiting the exposure of fewer subjects to drugs and procedures.

### Toxicology

Twenty‐eight day toxicology in rats and dogs for Investigational New Drug filing was performed by Charles Rivers (UK). Reproductive toxicology in rabbits was performed by Charles Rivers (Canada). Pharmacokinetic and/or toxicological doses ranged from 5 to 1000 mg·kg^−1^ p.o. in Sprague Dawley male and female rats (*n* = 6‐26 per group), 50–200 mg·kg^−1^ p.o. in male and female Beagle dogs (*n* = 6 per group) and 100–750 mg·kg^−1^ p.o. in female New Zealand white rabbits (*n* = 9 per group). Results are expressed as mean ± SD. The sample size was based on experience from previous similar toxicology studies with other compounds to obtain adequate safety, tolerability and pharmacokinetics data to achieve the objectives of the study.

### Electrically‐evoked contraction of the mouse vas deferens

Vasa deferentia were obtained from outbred MF‐1 mice and mounted vertically in a 4 mL organ bath at an initial tension of 0.5 g. The baths contained Mg^2+^‐free Krebs solution (NaCl 118.2 mM, KCl 4.75 mM, KH_2_PO_4_ 1.19 mM, NaHCO_3_ 25.0 mM, glucose 11.0 mM and CaCl_2_·6H_2_O 2.54 mM), which was kept at 35–36°C and bubbled with 95% O_2_ and 5% CO_2_. Isometric contractions were evoked by stimulation with 0.5 s trains of three pulses of 110% maximal voltage (train frequency 0.1 Hz; pulse duration 0.5 ms) through a platinum electrode attached to the upper end and a stainless steel electrode attached to the lower end of each bath (Ross *et al.,* 2001). Contractions were monitored by a computer using a data recording and analysis system as described previously (Ross *et al.,* 2001). After placement in an organ bath, each tissue was subjected to a stimulation‐free period of 10 min and then stimulated for 10 min. Tissues were then subjected to alternate periods of stimulation (5 min) and rest (10 min) until consistent twitch amplitudes were obtained. This equilibration procedure was followed by a stimulation‐free period of 25 min. Tissues were then stimulated for 5 min after which the first agonist addition was made and stimulation continued until the end of the experiment.

### Receptor binding

Receptor binding/agonism activity of 10 μM VSN16R, plus positive controls, was performed on cell lines transfected with human cannabinoid ***CNR1*** and ***CNR2*** and large number of other receptors and transporters and was performed by Cerep SA, (Poitiers, France); Chantest Inc. (Cleveland Ohio), DiscoveRx (Birmingham, UK); Multispan Inc., (Hayward, CA, USA); and MDS Pharma services (Taipei, Taiwan).

### Methoxamine‐evoked contraction of rat mesenteric artery

Adult male Wistar rats (200–350 g) were killed by cervical dislocation and the third‐order branches of the superior mesenteric artery, which provides the blood supply to the intestine, were removed and cleaned of adherent tissue. Segments (2 mm in length) were mounted in a Mulvany–Halpern‐type wire myograph (Model 610 M; Danish Myo Technology, Aarhus, Denmark) and maintained at 37°C in gassed (95% O_2_/5% CO_2_) Krebs–Henseleit solution of the following composition (mM): 118 NaCl, 4.7 KCl, 1.2 MgSO_4_, 1.2 KH_2_PO4, 25 NaHCO_3_, 2 CaCl_2_ and 10 D‐glucose, as previously described (Ho and Randall, 2007). Vessels were equilibrated and set to a basal tension of 2 to 2.5 mN. The integrity of the endothelium was assessed by pre‐contracting the vessel with 10 μM methoxamine (an α_1_‐adrenoceptor agonist), followed by relaxation with 10 μM carbachol (a muscarinic acetylcholine receptor agonist); vessels showing relaxations of greater than 90% were designated as endothelium‐intact. When the endothelium was not required, it was removed by rubbing the intima with a human hair; carbachol‐induced relaxation of less than 10% indicated successful removal. After the test for endothelial integrity, vessels were left for 30 min and then pre‐contracted with 10 μM methoxamine. This was followed by construction of a cumulative concentration–relaxation curve to VSN16R (10 nM–1 μM). To investigate the relaxation mechanisms of VSN16R, calcium‐activated, potassium channel blockers (apamin, charybdotoxin or iberiotoxin) were incubated with the vessels either alone or in combination before construction of the concentration–response curves to VSN16R. The tension generated by methoxamine in control vessels was 9.4 ± 0.6 mN, as compared with 10.4 ± 2.5 mN in endothelium‐denuded vessels, or 10.5 ± 0.5 mN in the presence of calcium‐activated, potassium channel inhibitors. Most experiments were performed in matched vessels; effects of blockers or endothelial removal were compared with the control responses obtained in separate vessels of the same rats. All relaxant responses are expressed as percentage relaxation of the tone induced by methoxamine. Values are given as mean ± SEM. Statistical comparisons of concentration‐dependent responses were made by two‐way ANOVA using Prism 4 (GraphPad Software, Inc, San Diego, CA, USA).

### Patch clamp of calcium‐activated potassium channels recording in human cells

The human EA.hy926 endothelial cells (ATCC, Manassas, USA) were grown in DMEM containing 10% FCS and 1% HAT (5 mM hypoxanthine, 20 μM aminopterin and 0.8 mM thymidine) and were maintained in an incubator at 37°C in 5% CO_2_ atmosphere (Bondarenko *et al.,* 2013). Human HNC‐2 neuronal cells were obtained from ATCC. For experiments, cells were plated on glass coverslips. Single‐channel recordings were obtained from excised inside‐out membrane patches in symmetrical solutions using the patch‐clamp technique. All patch‐clamp experiments were performed at room temperature with the use of a water hydraulic micromanipulator (WR‐6, Narishige, Japan). Patch pipettes were pulled from glass capillaries using a Narishige puller (Narishige Co. Ltd, Tokyo, Japan) that was fire polished and had a resistance of 3–5 MΩ for whole‐cell recordings and 5–7 MΩ for single‐channel recordings. The pipettes were filled with 140 mM KCl, 10 mM HEPES, 1 mM MgCl_2_, 5 mM EGTA and 4.9 M CaCl_2_ buffered to pH 7.2 by adding KOH. Cells were super‐perfused with a bath solution containing 140 mM NaCl, 5 mM KCl, 1.2 mM MgCl_2_, 10 mM HEPES, 10 mM glucose and 2.4 mM CaCl_2_. Following gigaseal formation, bath solution was switched to the following: 140 mM KCl, 10 mM HEPES, 1 mM MgCl_2_, 5 mM EGTA and a designated free Ca^2+^ concentration, which was adjusted by adding different amounts of CaCl_2_ buffered to pH 7.2. (Bondarenko *et al.,* 2011; Bondarenko *et al.,* 2013). Single‐channel activity was expressed as *N*Po, where *N* represents the number of functional ion channels in the membrane patch and Po represents the open channel probability determined from idealized traces (Bondarenko *et al.,* 2011). *N*Po was obtained from ≥20 s of continuous recording under each experimental condition. The effect of VSN16R was estimated after 3 min of continuous exposure to VSN16R‐containing solution. Data were analysed by ANOVA, and statistical significance was evaluated using Scheffés *post hoc* test of the Prism 5 software for Windows (GraphPad Software).

### Pharmacokinetics

The stability of compounds when subjected to hepatic and plasma degradation was assessed *in vitro* by Inpharmatica, Cambridge, UK. Compounds (1 μM) were incubated with either pooled mouse liver microsomes (0.1 mg protein mL^−1^) or pooled mouse plasma at 37°C for 0, 5, 10, 20 and 40 min before termination with acetonitrile containing warfarin as an analytical internal standard. Samples were centrifuged, and the resultant supernatant analysed for parent compound. The mass responses at baseline were taken as the 100% reference values against which the disappearance of the compound was measured. The natural log of the % remaining values was used to generate linear plots of disappearance of the compounds. Half‐life values were calculated from the slope of these plots. Hepatocyte metabolism was assessed by Cyprotex, Macclesfield, and 10 μM VSN16R was incubated with primary mouse, rat, monkey (Macaque) and human hepatocytes for 1 h, and the presence of metabolites was assessed using liquid crystallography mass spectroscopy. The stability of VSN16R was assessed *in vivo* by Inpharmatica, Cyprotex and Charles Rivers, Cambridge. Blood (plasma) samples were obtained prior to and 5 min to 24 h after drug administration of 2–5 mg·kg^−1^ i.v. or 5 mg·kg^−1^ p.o. VSN16R into outbred mice and rats (*n* = 3 per time point). In addition, plasma, brain and spinal cord samples were obtained from ABH with spastic EAE. Immediately after blood collection, the brain and spinal cord were removed and then stored at −20°C prior to assay. Tissue samples were weighed, homogenized and centrifuged, and the lysates generated. Brain lysates and plasma were assayed by LC–MS methods by Inpharmatica/Charles Rivers, Cambridge, UK. This was also performed using a Waters Xevo TQ or API4000, AB Sciex mass spectrometer using spiked samples as standards. PK Solutions Software (Summit Research Services, Montrose, CO, USA), AB Sciex Analyst software combined with Thermo Fisher Scientific Watson LIMS processing and reporting software or WinNonLin pharmacokinetic or PK solutions 2.0 software (Summit Research Services, Montrose CO, USA) were used to produce PK parameters. Data are expressed as mean ± SEM. The detection limit of VSN16R was 2 ng·mL^−1^ in plasma and brain and 14 ng·mL^−1^ in spinal cords. The detection limit of VSN22R was 2 ng·mL^−1^ in plasma and 0.1 ng·mL^−1^ for VSN44R in plasma. Pharmacokinetics in toxicological samples in rats (*n* = 6 per group), dogs (*n* = 6 per group) and humans (*n* = 6 per group) were performed by Charles Rivers (Edinburgh, UK).

### Open‐field activity monitoring

Motor activity was assessed using 27.9 × 27.9 cm open field activity monitor chambers and computer software (Med Associates Inc, St. Albans, VT, USA.). This was typically performed in a darkened room. Recordings were initiated once the mouse entered the chamber and continued for 5 min. These chambers, allowing four simultaneous recordings of individual mice and were fitted with infrared beams that could detect movement in the X, Y planes. The total distance travelled (cm) was recorded. Results are expressed as mean ± SEM. These were analysed by Student's paired *t*‐test or a one‐way ANOVA with Bonferroni *post hoc* test, using SigmaStat V3/Sigmaplot V9 or V11 software (Systat Ltd Hounslow, UK. Al‐Izki *et al.,* 2012).

### Temperature testing

Temperature of animals was monitored by using a thermocouple placed under the hindlimb, and the temperature was allowed to equilibrate and was recorded once it failed to increase further (Pryce *et al.,* 2014). These recordings were assessed 20 min following administration of compounds. Results are expressed as mean ± SEM, and were analysed by Student's *t*‐test or a one‐way ANOVA with Bonferroni *post hoc* test using Sigma/StatV3/Sigmpaplot V9 or V11 software (Pryce *et al.,* 2014).

### Induction of spasticity

Disease was induced in ABH mice by immunization with spinal cord homogenate in Freunds adjuvant as described previously and included data relevant to the ARRIVE guidelines related to EAE induction (Al‐Izki *et al.,* 2012; Pryce *et al.,* 2014). Following the development of chronic relapsing EAE, spasticity typically developed after two to three relapses, about 80–100 days post‐induction onwards as a consequence of the accumulation of nerve damage (Baker *et al.,* 2000; Al‐Izki *et al.,* 2012). This was assessed in non‐anaesthetized, awake animals during remission from active paralytic episodes by the force required to bend the hind limb to full flexion against a custom‐built strain gauge, following extension of the limb prior to measurement. This cannot be used on limbs that are flexed, which were not measured. Animals with visible spasticity were used as spasticity became apparent. The data were analysed using Spike 2 software (Cambridge Electronic Design, UK), and a mean score for each limb at each time point was calculated, and forces were converted to Newtons (Baker *et al.,* 2000; Pryce *et al.,* 2014). Animals were entered into the study following the development of visible spasticity. These mice were randomly assigned to treatment group by the person administering the compounds or vehicle, and mice were assessed in a blinded fashion by a different assessor. Data were analysed blinded to treatment. Each group contained a minimum of five different animals. This would provide 80% power to detect a 25% change in limb (*n* = 10) stiffness at the *P* < 0.05 level, as reported previously (Pryce *et al.,* 2014). The results represent the mean ± SEM resistance to flexion force (*N*) or individual limbs, which were compared using one‐way repeated measures ANOVA incorporating a Student–Newman–Keuls *post hoc* test or infrequently paired *t*‐tests using SigmaStat V3/Sigmaplot V9 or V11 software (Pryce *et al.,* 2014). Resistance forces of limbs below 0.15 N were typically excluded from analysis, as this is below the normal range on non‐spastic limbs (Baker *et al.,* 2000) consistent with a pre‐defined protocol. Differences were compared with baseline using repeated measures ANOVA rather than comparison of group means, because there was such marked differences in the severity of spasticity in individual limbs, resulting in variable baseline group means, as shown previously (Baker *et al.,* 2000). Therefore, to assist in comparison, data were sometimes also expressed as percentage change ± SEM from baseline.

### Electrophysiology

Animals with spasticity were anaesthetized using 50 mg·kg^−1^ ketamine (Modol *et al.,* 2014) and 1 mg·kg^−1^ dexmedetomidine/medetomidine according to veterinary advice. The experiment was terminated, without recovery, while the animals were still anaesthetized. A drop of 2% hyroxypropyl methylcellulose was added to the eyes to keep them moist. The animal was secured in a stereotactic frame, and body temperature was maintained at 37°C with a small heating plate with a built‐in resistance temperature detector sensor connected to a direct current temperature controller linked to an anal thermocouple. The legs and nape of the neck were shaved, and the sciatic nerve in the upper thigh was surgically exposed. The animal, at the nape of the neck, and heating pads were earthed using electrodes. The animal was covered to conserve heat. The stimulating electrode was placed under the sciatic nerve and sealed with white petroleum jelly. Stimuli of 100 ms duration constant current were delivered to the sciatic nerve using a Neurolog NL800 (Digitimer), at a rate of 0.2 Hz, and the current increased until a convincing recording of both M‐wave (stimulus from electrode to muscle) and H‐wave (stimulus from the electrode to the spinal reflex arc and from motor nerves to muscle approximately 7 ms after the M wave (Modol *et al.,* 2014) were generated in each sweep). The current required varied from preparation to preparation but was substantially less than that needed to elicit supramaximal nerve activation (current range ≈1 mA; Modol *et al.,* 2014) Differential motor unit recording from anterior tibialis was achieved using a Neurolog AC preamplifier and headstage (Digitimer, Welwyn Garden City, UK). Two monopolar 25 gauge needle recording electrodes were inserted into the muscle at either end, the signal was amplified, band‐pass filtered between 200 Hz and 1 KHz, digitized by a 1401 plus interface (at 3 KHz) and then displayed and saved by a computer running Signal software (Cambridge Electronic Design). VSN16R 30 mg·kg^−1^ or 0.1 mL PBS was injected i.v. *via* the tail vein. This route was selected as it could be administered without further movement of the animal or disturbance of the recording electrodes. The high starting dose was selected based on the amount of VSN16R that could easily be dissolved in saline to maximize duration, as a starting point. The response was recorded for 12 min, although once detected the H response was maintained without attenuation for over a 30 min. The amplitude (mV) was measured and compared with that at the time of injection, taken as 100%, rather than a mean response, due to variability in the current required to elicit the maximal H response.

### RNA extraction and sequencing

Three EAE mice demonstrating spasticity and three age‐matched control mice were killed, and the spinal cord tissue was removed, snap‐frozen and stored at −80°C. These samples were analysed by RNAseq as described previously (Sevastou *et al.,* 2016). Frozen tissue was disrupted in TRIzol® Reagent on ice, using a rotor‐stator homogenizer. Following 5 min incubation at room temperature, chloroform was added to the samples, which were shaken, left to rest and then centrifuged at 12 000 g for 15 min. The resulting upper aqueous phase was washed with 70% ethanol, mixed well and loaded on an RNeasy column. Thereafter, the Qiagen RNeasy® Mini Kit protocol was followed to extract and purify mRNA. The mRNA integrity was assessed by microfluidic capillary electrophoresis using the Agilent 2100 Bioanalyzer. All samples had a 260/280 ratio > 1.8 with RNA integrity number > 9. RNA sequencing was performed at the UCL Genomics facility (UCL Institute of Child Health, London) using the Illumina NextSeq 500 platform. The FASTQ files generated for each sample were aligned to the UCSC Mus musculus HG19 reference genome using the TopHat2 software (Illumina). Downstream analysis of these alignments was performed using Cufflinks software (Illumina). Cufflinks computes normalized fragments per kilobase of exon per million fragments mapped, which reflect the expression levels of each mRNA molecule. The readings were mapped to a total of 23 352 genes and 30 608 transcripts. The statistical analysis resulted in *P* values corrected for multiple testing with a default false discovery rate of *q* < 0.05 (Sevastou *et al.,* 2016).

### Western blotting

Mouse spinal cord whole tissue was homogenized in RIPA buffer (150 mM NaCl, 1.0% IGEPAL CA‐630, 0.5% sodium deoxycholate, 0.1% SDS and 50 mM Tris at pH 8.0), supplemented with protease and phosphatase inhibitors (Sigma, Poole, Dorset). Similarly, EA.hy926 cells were lysed in RIPA buffer supplemented with protease and phosphatase inhibitors. The proteins were resolved on SDS‐PAGE and transferred onto Immobilon‐P‐PVDF membranes. Membranes were washed, blocked for 1 h in blocking buffer (5% non‐fat dried milk) and incubated in blocking buffer with primary antibody using rabbit polyclonal antibodies against mouse/human ***KCNMA1*** (APC‐021, previously validated by lack of activity in big conductance calcium‐activated potassium channel *Kcnma1*‐deficient mice and APC‐151 antibody) and *KCNMB4* (APC‐061 antibody, previously validated by lack of activity in *Kcnmb4*‐deficient mice despite detecting multiple isoforms), which were purchased from Alomone Labs, Jerusalem Israel, whose website reports supporting literature concerning characterization of the antibodies. These were incubated overnight at 4°C at 1:200 dilution or mouse monoclonal antibodies incubated for 2 h at room temperature (anti‐β‐actin 1:2000, Santa Cruz BioTech sc‐101663). After repeated washes in Tris buffered saline with Tween 20 (T‐TBS; 10 mm Tris–HCl, 150 mm NaCl and 0.5% Tween 20, pH 7.4), membranes were incubated with horseradish peroxidase‐conjugated secondary antibody (anti‐rabbit immunoglobulins 1:4000, Santa Cruz BioTech sc‐2004 or anti‐mouse immunoglobulins 1:1000, ThermoFisher. SA1‐100) in T‐TBS for 1 h. Blots were developed by enhanced chemiluminescence detection.

### Data and statistical analysis

The data and statistical analysis comply with the recommendations on experimental design and analysis in pharmacology (Curtis *et al.*, 2015). Most experiments contained a minimum of 5 animals or individual samples, consistent with sample size calculations and experience from previous studies, as indicated. Group sizes of less than *n* = 5 were used in some pharmacokinetic studies, based on a minimum numbers approach. However, rodent pharmacokinetic studies were replicated in additional experiments. The statistical tests used are reported in the methodological subsections. If this involved parametric analysis, this included analysis of normality and equality of variance. The level of probability deemed to constitute the threshold for statistical significance was *P < 0.05*.

### Chemicals

The production and synthesis of VSN16 (318 Da) and the VSN16R and VSN16S enantiomers and VSN22 were as described previously (Hoi *et al.,* 2007). VSN44 was also synthesized (Supporting Information Methods S1). These compounds were additionally synthesized to either Good Manufacturing Practice or Good Laboratories Practice Standards by Sygnature Chemical Services (Nottingham, UK), Park Place Research (Cardiff, UK) or Dalton Chemical Laboratories Inc. (Toronto, Canada). VSN16R for human use was formulated in 25 and 100 mg gelatin capsules by Dalton Chemical Laboratories. R(+)**WIN55‐212** was purchased from Tocris Ltd. (Bristol, UK) and ±**baclofen** was purchased from Sigma Aldrich Ltd (Poole, UK). 1′, 1′‐dimethylheptyl‐M8‐tetrahydrocannabinol‐11‐oic acid (CT3) was supplied by Atlantic Ventures Inc. (New York, USA) (Pryce *et al.,* 2014). **BMS‐204352**; **NS‐1619**, NS‐11021, **paxilline** and **CNQX** were purchased from Tocris Ltd or Sigma (Poole, UK). Compounds were dissolved in water; saline or DMSO (WIN55‐212) or ethanol prior to dilution with cremaphor (Sigma) and PBS (1:1:18). These drugs were delivered *via* the p.o., i.p. or i.v. routes at less than 5 mL·kg^−1^, typically 0.1 mL in mice and 0.5 mL in rats. Although VSN16R is water soluble (>30 mg·mL^−1^), for higher concentrations (200 mg·mL^−1^), it was dissolved in 20% polypropylene in water.

### Nomenclature of targets and ligands

Key protein targets and ligands in this article are hyperlinked to corresponding entries in http://www.guidetopharmacology.org, the common portal for data from the IUPHAR/BPS Guide to PHARMACOLOGY (Southan *et al.,* 2016), and are permanently archived in the Concise Guide to PHARMACOLOGY 2015/16 (Alexander *et al.,* 2015a,b,c,d).

## Results

### VSN16 is a novel anti‐spastic agent

**Figure 1:**
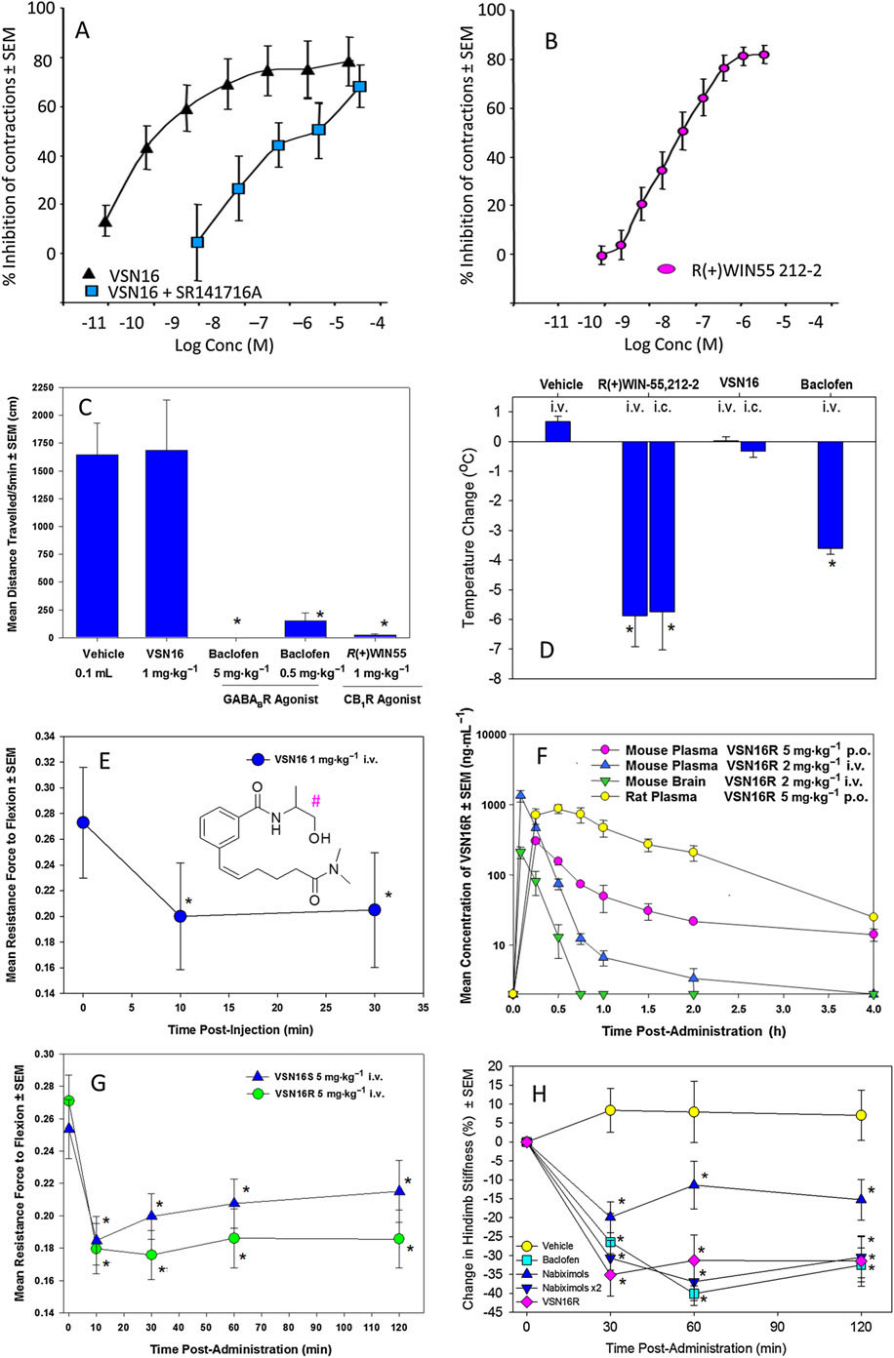
VSN16 inhibits spasticity without sedation. (A, B) Inhibition of electrically evoked contraction of the mouse vas deferens following incubation with various doses of (A) VSN16 with or without pre‐incubation of 31.6 nM SR141617A, (B) R(+)WIN55,212; *n* = 5–6 replicates. (C, D) Lack of sedative effect of 1 mg·kg^−1^ VSN16 in ABH mice compared with 1 mg·kg^−1^ R(+)WIN55,212 (*n* = 5) or 5 mg·kg^−1^ i.v. baclofen (*n* = 4). The results represent the mean ± SEM. (C) Hypomotility in a 27 × 27 cm open field chamber 30 min after i.v. drug administration and (D) hypothermia 20 min after drug administration, *via* the i.v. or intracerebral (i.c.) route, in wild‐type ABH mice or CB_1_ receptor‐deficient mice (*n* = 4 animals per group) except baclofen (*n* = 10 animals).(E) Inhibition of spasticity measured as the resistance to hindlimb flexion against a strain gauge in visibly spastic ABH mice, showing mean ± SEM following injection of 1 mg·kg^−1^ i.v. VSN16 racemate; *n* = 6 animals, *n* = 9 limbs. *Significantly different compared with baseline. VSN16 structure in the inset with chiral centre (#) indicated. (F) Pharmacokinetics of VSN16R in outbred mice and rats following i.v. or p.o. administration. Data represent mean ± SEM; *n* = 3 male animals per group. (G, H) Inhibition of spasticity measured as the resistance to hindlimb flexion against a strain gauge in visibly spastic ABH mice; (G) 5 mg·kg^−1^ i.v. VSN16R (*n* = 7 animals, *n* = 14 limbs) or VSN16S (*n* = 7 animals, *n* = 13 limbs). (H) The % change in resistance to hindlimb flexion from baseline following vehicle (*n* = 8 animals, *n* = 14 limbs), 5 mg·kg^−1^ i.v. VSN16R (*n* = 7 animals, *n* = 14 limbs), 5 mg·kg^−1^ i.v. baclofen (*n* = 6 animals, *n* = 10 limbs) or 5 mg·kg^−1^ i.v. (*n* = 8 animals, *n* = 13 limbs) or 10 mg·kg^−1^ i.v. (*n* = 6 animals, *n* = 11 limbs) using botanical drug substances within medicinal cannabis (Hilliard *et al.,* 2012). These were undertaken as separate experiments. *Significantly different compared with baseline.

Following the synthesis of VSN16, the affinity of VSN16 (racemate) for the CB_1_ receptor was first assessed by measuring the relaxation of electrically‐evoked contractions of the vas deferens (Ross *et al.,* 2001) This is a standard, tissue‐based assay used to detect cannabinoid receptor activity (Ross *et al.,* 2001) but assesses autonomic neurotransmission for a wide range of receptors, *via* actions on nerves originating from pelvic and lumbar/sacral ganglia (Burnstock and Verkhratsky, 2010). VSN16 exhibited potent inhibitory activity with an EC_50_ in the low nM (~10 nM) range (Figure 1A). This compared favourably with the potent CB_1_/CB_2_ receptor agonist *R*(+)WIN55–212 (Figure 1B), although the slope of the VSN16 dose–response curve (Figure 1A) appeared to be different, possibly suggesting activity at a distinct target or targets. VSN16R did not affect β γ, methylene ATP‐induced contractions in electrically unstimulated vasa deferentia (Supporting Information Figure S2), indicating that the action of VSN16R was not directly on the muscle. Although VSN16 was at least as potent as *R*(+)WIN55‐212 (Figure 1A, B), it failed to induce visible signs of sedation at 1 mg·kg^−1^ i.v. and did not induce hypomotility (Figure 1C) or hypothermia (Figure 1D, which are indicative of central cannabimimetic effects in rodents (Varvel *et al.,* 2005). This contrasts to the significant sedative effects induced by *R(+)*WIN55‐212 (Figure 1C, D), which are absent in CB_1_ receptor‐deficit mice (Pryce *et al.,* 2014). Whilst the action of VSN16 could be antagonized following pretreatment of the vas deferens with **SR141716A**, a CB_1_ receptor antagonist (Figure 1A), when VSN16 was injected directly into the CNS, it also failed to induce cannabimimetic effects (Figure 1D), clearly indicating that VSN16 was neither a global nor a CNS‐excluded CB_1_ receptor agonist *in vivo* at the doses tested, and thus was mediating its effects by another SR141716A‐sensitive mechanism.

In MS, spasticity is measured *via* subjective assessments that are either physician‐assessed, such as the modified Ashworth Scale, or patient‐assessed numerical rating scales (Shakespeare *et al.,* 2003; Novotna *et al.,* 2011; Wininger *et al.,* 2015). These are limited compared with objective measures and may be poorly responsive to treatments (Shakespeare *et al.,* 2003; Novotna *et al.,* 2011; Wininger *et al.,* 2015). Although spasticity in mice was visually evident, it was objectively and quantitatively measured using a strain gauge to detect limb stiffness (Baker *et al.,* 2000). Interestingly, it was found that 1 mg·kg^−1^ i.v. VSN16 could significantly inhibit spasticity within a few minutes of administration (Figure 1E) and occurred in the absence of sedative effects (Figure 1C, D). As VSN16 is chiral, both the R and S enantiomers were made and VSN16R (EC_50_ = 10 nM) was slightly more active than VSN16S (EC_50_ = 37 nM) in the mouse vas deferens assay, as was also subsequently seen in the vasorelaxation of rat mesenteric arteries, which is another standard tissue‐based assay used to measure vascular cannabinoid receptor activity (VSN16R EC_50_ = 110 nM, VSN16S = 140 nM; Hoi *et al.,* 2007). However, when the *in vivo* pharmacokinetic responses were assessed in both outbred mice and rats (Figure 1F), the clearance of VSN16R administered *via* the i.v. route was relatively fast (half‐life of 7–11 min). This demonstrated CNS penetration with a plasma : brain ratio at maximum concentration (C_max_) of 0.16 in healthy mice (Figure 1E). It was found that VSN16R appeared slightly more active than VSN16S in spasticity when tested at 5 mg·kg^−1^ i.v. (Figure 1G) and, therefore, subsequent studies focused on VSN16R. VSN16R 5 mg·kg^−1^ i.v. could induce comparable inhibition of spasticity to that of 5 mg·kg^−1^ i.v. baclofen (Figure 1H), although this dose of baclofen was associated with significant sedation (Figure 1C, D). Likewise, VSN16R appeared to be as potent as 5–10 mg·kg^−1^ i.v. (sedative) doses of the botanical drug substances in nabiximols/medicinal cannabis (Figure 1H; Hilliard *et al.,* 2012). Therefore, while VSN16R may be no more potent than current anti‐spastic agents, it lacked their sedative effects, which limits compliance and early use in spasticity in MS.

### VSN16R is an orally active, anti‐spastic agent

In contrast to the i.v. route, the delivery of 5 mg·kg^−1^ *via* the p.o route showed a longer elimination half‐life in rodents [89 min in mice and 43 min in rats (Figure 1E)]. Oral absorption was rapid, and C_max_ was detected within 15 min from administration in mice indicating good absorption from the gastrointestinal tract, and good p.o. bioavailability (22–40% mouse‐rat, respectively, after 5 mg·kg^−1^ p.o.) was evident (Figure 1F). Doses of up to 30 mg·kg^−1^ i.v. VSN16R in PBS failed to show any visible evidence of sedation in mice (*n* = 5 animals), and repeated daily treatment of 1 g·kg^−1^ p.o. in water to mice for 5 days failed to show any overt behavioural effects or toxicity and no drug‐induced weight loss (29.0 ± 2.0 g vs. 30.2 ± 1.1, *n* = 3 mice). Oral VSN16R was also very‐well tolerated in rats, and behavioural testing of rats treated with 120 mg·kg^−1^ p.o. (*n* = 6) did not display any adverse behavioural responses in Irwin tests (Roux *et al.,* 2005), which assesses over 30 largely, neurological and movement, behavioural outcomes (Supporting Information Table S1).

**Figure 2:**
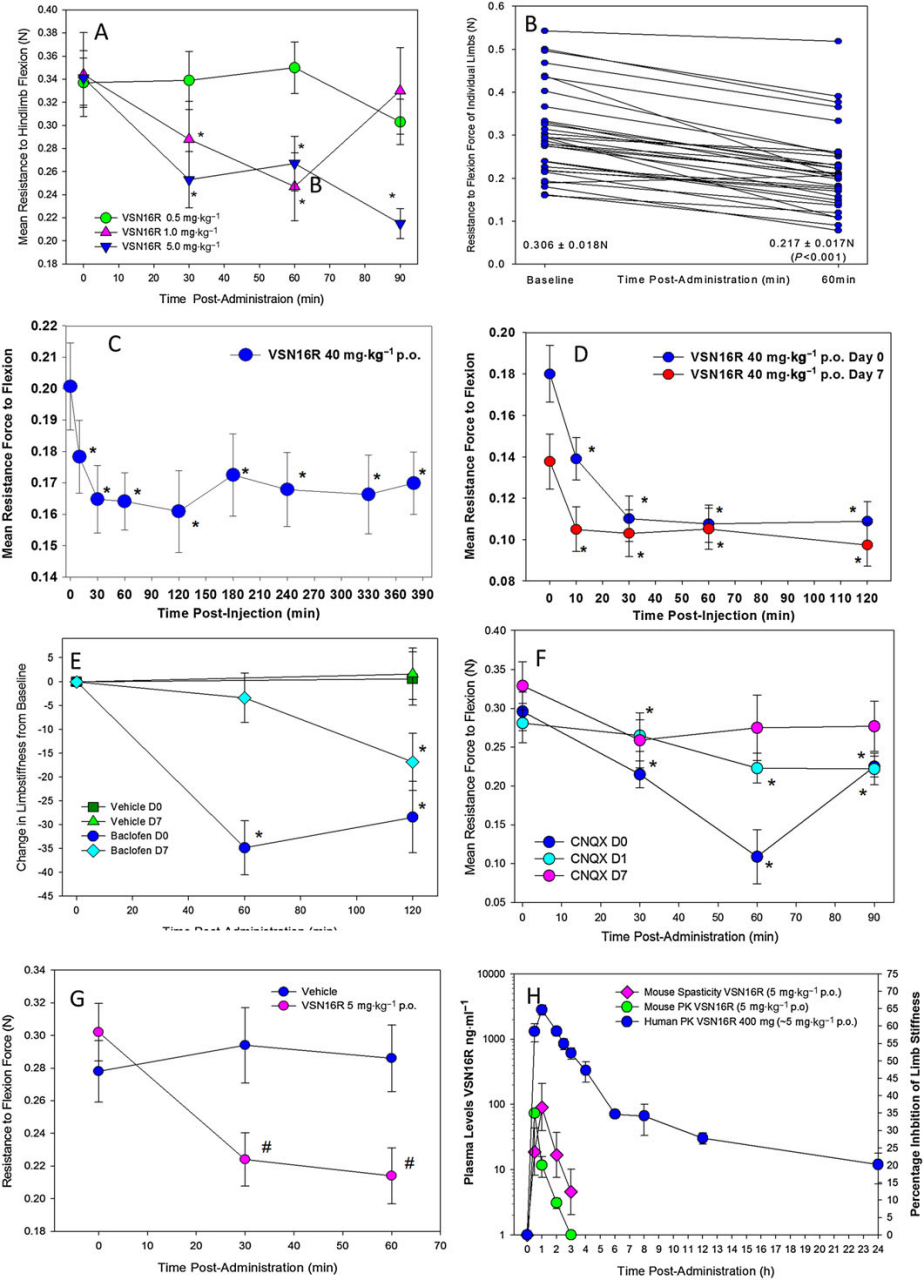
Oral VSN16R inhibits spasticity. Inhibition of spasticity measured as the resistance to flexion against a strain gauge in visibly spastic ABH mice showing the force required for flexion. Animals received p.o. (A) 0.5 mg·kg^−1^ (*n* = 10 animals; *n* = 20 limbs), 1 mg·kg^−1^ (*n* = 10 animals *n* = 18 limbs) or 5 mg·kg^−1^ p.o. VSN16R in water (*n* = 10 animals, *n* = 19 limbs). (B) The change in resistance to flexion forces of individual limbs from baseline to 60 min following 5 mg·kg^−1^ p.o. administration (*n* = 17 animals, *n* = 32 limbs). (C) Effect of 40 mg·kg^−1^ VSN16R (*n* = 8 animals, *n* = 15 limbs). (D) Repeated daily administration of 40 mg·kg^−1^ with measurement on the first and eighth day of treatment (*n* = 8 animals *n* = 15 limbs). (E) Repeated daily administration of vehicle (*n* = 9, *n* = 18 limbs) or 5 mg·kg^−1^ p.o. in baclofen in water (*n* = 7 animals, *n* = 14 limbs), where there was no difference at baseline between days 0 and 7. (E) Repeated daily 25 mg·kg^−1^ CNQX s.c. (*n* = 5 animals, *n* = 10 limbs). *Significantly different compared with baseline. (F) Comparison of 5 mg·kg^−1^ p.o. VSN16R in water (*n* = 17 animals) and vehicle‐treated animals (*n* = 17 animals) using a single mean combined from both limbs, rather than analysis of individual limbs. #Significantly different compared with vehicle‐treated animals. (F) Plasma levels of VSN16R following p.o. delivery of 5 mg·kg^−1^ VSN16R to spastic ABH mice with EAE or 400 mg VSN16R (~5 mg·kg^−1^) p.o. in healthy humans; *n* = 6.

The effect of p.o. VSN16R on spasticity was analysed in ABH mice with EAE (Figure 2). Whilst 0.5 mg·kg^−1^ p.o. VSN16R failed to produce rapid muscular relaxation and inhibition of spasticity (Figure 2A), significant therapeutic activity was evident within 10 min following administration of 1 mg·kg^−1^ and 5 mg·kg^−1^ VSN16R p.o. (Figure 2A). Therefore, VSN16R has over 1000‐fold therapeutic window. Despite the marked variability in spasticity in individual limbs (Figure 2B), which could influence group means depending on which animals were assessed (Figure 2C, D), it was evident that most limbs showed a significant level of relaxation irrespective of the degree of initial spasticity when treated with 5 mg·kg^−1^ p.o. VSN16R (*n* = 17 animals, 32 = limbs; Figure 2B). The duration of activity was dose‐dependent, and inhibition of spasticity lasted for over 6 h following a single dose of 40 mg·kg^−1^ p.o. (Figure 2C). Furthermore, the therapeutic effect was sustained after repeated daily administration of 40 mg·kg^−1^ VSN16R p.o. for 7 days (Figure 2D). This suggested a lack of significant receptor desensitization and even some cumulative benefit, as following repeated administration there was significantly reduced spasticity compared with starting values 7 days earlier (Figure 2D; *n* = 15 limbs from eight mice). However, spasticity returned to baseline levels following cessation of treatment (resistance to flexion force baseline 0.174 ± 0.033 N) 2 weeks after cessation of weekly VSN16R treatment (0.157 ± 0.045 N [mean ± SEM] *n* = 10 limbs from six animals. Two animals were reached humane endpoints after termination of VSN16R dosing). Repeated daily vehicle failed to influence the degree of spasticity (Figure 2E). However, in contrast to the lack of desensitization with repeated VSN16R (Figure 2D), repeated 5 mg·kg^−1^ p.o. baclofen (GABA_B_ agonist, Figure 2E) and 25 mg·kg^−1^ s.c. CNQX (AMPA/kainate glutamate receptor antagonist, Figure 2F) showed inhibitory responses that diminished over time (Figure 2E, F). This was indicative of receptor desensitization and supports the need to dose‐escalate baclofen when used in humans (Shakespeare *et al.,* 2003). Furthermore, in contrast to the loss of muscle tone in healthy animals that can occur following sedation (Supporting Information Figure S3), 40 mg·kg^−1^ p.o. caused no significant loss of muscle tone in normal animals [9.1 ± 6.3% change (increase) from baseline *n* = 6 animals compared with a significant 17.8 ± 5.3% drop (*n* = 8 animals; compared with baseline using repeated measures ANOVA) in visibly spastic animals]. In this analysis (Figure 2A–F), each limb was treated as the unit of assessment, to encompass the marked variability of spasticity in different limbs (Figure 2B) and offers 3Rs value to limit animal use (Baker *et al.,* 2000). However, to address concerns of pseudo‐replication *via* analysis of both limbs, when only one mean result per animal was assessed, again, 0.1 mL vehicle failed to influence spasticity (*P* > 0.05, *n* = 17 animals) and again, 5 mg·kg^−1^ p.o. significantly (at 30 min, 60 min) inhibited spasticity (*n* = 17 animals) when compared with baseline (Figure 2G). Furthermore, whilst the mean group score (±SD) in vehicle‐treated animals (0.278 ± 0.078 N) was no different from the baseline of animals treated with 5 mg·kg^−1^ p.o. (0.302 ± 0.073 N), animals were significantly less spastic 30 min (0.224 ± 0.067 N) and 60 min (0.214 ± 0.071 N) following treatment with 5 mg·kg^−1^ p.o. compared with vehicle‐treated animals (Figure 2G).

The Hoffman (H) reflex, where the spinal reflex is highly exaggerated due to over excitation/loss of descending inhibitory tone following the CNS damage that causes spasticity (Matthews, 1966; Baker *et al.,* 2012) was assessed (Supporting Information Figure S3). Although it is often used in experimental spasticity studies (Modol *et al.,* 2014), electrophysiology may not always detect anti‐spastic effects in humans and does not always yield consistent results (Leocani *et al.,* 2015). Whilst the H reflex following stimulation of the sciatic nerve and recording from the anterior tibialis muscle (Modol *et al.,* 2014) could be variably detected and modified by VSN16R in some animals, this was not consistent (Supporting Information Figure S3). Furthermore, this approach was considered of limited value as it was clear that the ketamine (NMDA antagonist) and medetomidine/dexmedetomidine hydrochloride (α_2_‐adrenoceptor agonist) anaesthetics typically used (Modol *et al.,* 2014), inhibited physical spasticity, and importantly, the anaesthetics were found to block the mechanism of action of VSN16R (Supporting Information Figure S3). Furthermore, the animals, which are severely neurologically affected could not tolerate the anaesthetic procedure, unlike healthy mice, and it often caused them to die. Therefore, the approach was terminated on ethical grounds.

It was clear from pharmacokinetic studies in mice that there was over 15% CNS penetration with VSN16R, even at low doses (Figure 1F). Given the high doses of VSN16R that could be administered, it was evident that brain penetration of VSN16R was not inducing abnormal behavioural effects (Supporting Information Table S1), consistent with the direct CNS injection of VSN16R (Figure 1D). Furthermore, CNS penetration is increased during spastic EAE, due to blood–brain barrier dysfunction (Al‐Izki *et al.,* 2014). As such, a CNS‐excluded CB_1_ receptor agonist (10 mg·kg^−1^ i.v. CT3; Pryce *et al.,* 2014) induced significant cannabimimetic effects in spastic animals (2.3 ± 0.4°C temperature loss, 20 min after administration, *n* = 6 mice) at doses that did not affect healthy mice (0.0 ± 0.1°C, *n* = 5 mice). In addition, it was possible to detect VSN16R in spinal cords of chronic EAE animals (brains 4/4 mice, spinals cords *n* = 2/4 mice) compared with normal animals (brains *n* = 4/4 mice, spinal cords *n* = 0/4 mice), despite the lack of sensitivity of the assay, indicating that there is some additional lesional targeting of VSN16R during EAE, as has been seen previously with other compounds that are not fully CNS‐penetrant (Al‐Izki *et al.,* 2014). The duration of action showed a reasonably good correlation with the pharmacodynamics of plasma levels of VSN16R in ABH mice with EAE (Figure 2H). Similar plasma levels of drug could easily be achieved and exceeded with comparable doses of VSN16R in healthy humans (Figure 2F).

### Metabolism of VSN16R produces biologically active metabolites

**Figure 3:**
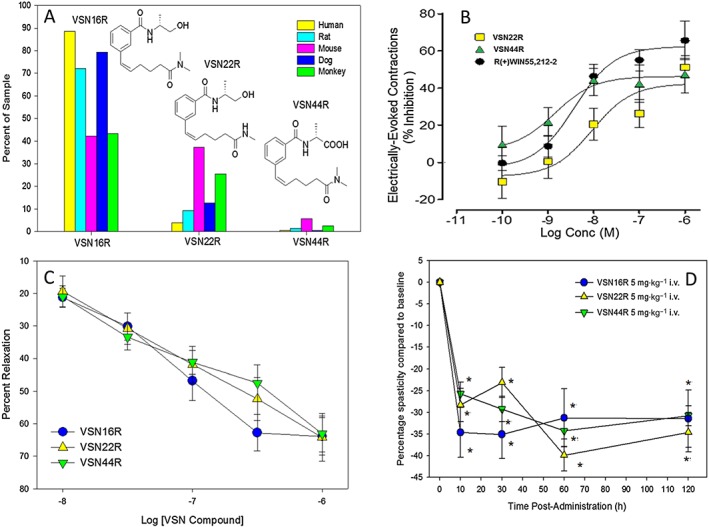
VSN16R produces active metabolites. (A) VSN16R was incubated with primary mouse rat, monkey and human hepatocytes and, 1 h later, the supernatants were assessed by liquid crystallography mass spectroscopy and two species, VSN22R and VSN44R, were identified, and the results expressed as percentage of the original starting material. Inset shows the structure of VSN22R and VSN44R. (B) Inhibition of electrically‐evoked contraction of the mouse vas deferens following incubation with various doses of VSN22R and VSN44R (*n* = 5 replicates). (C) VSN16R (*n* = 8 cultures), VSN22R (*n* = 6 cultures), VSN44R (*n* = 8 cultures)‐mediated relaxation of methoxamine‐evoked contraction in rat mesenteric arteries. (D) Inhibition of spasticity measured as the resistance to hindlimb flexion against a strain gauge in visibly spastic ABH mice, showing mean ± SEM following injection of 5 mg·kg^−1^ i.v. VSN16R (*n* = 7 animals, *n* = 14 limbs), VSN22R (*n* = 7 animals, *n* = 14 limbs) or VSN44R (*n* = 9 animals, *n* = 18 limbs). *Significantly different compared with baseline.

Pharmacokinetic studies have indicated rapid absorption of VSN16R in rodents (Figure 1F). Renal excretion accounted for less than 0.5% of the dose administered, and faecal elimination was approximately 1% of the dose in the first 12 h following p.o. dosing of rats (*n* = 3). VSN16R was stable (*t*_½_ >100 min which was the upper limit for assessment) in liver microsomes and plasma, compared with midazolam (*t*_½_ = 17 min) in liver microsomes and bisacodyl (*t*_½_ = 2 min) in plasma. VSN16R showed no significant inhibition of cytochrome p450 enzymes at 10 μM suggesting limited potential for drug–drug interactions (Supporting Information Table S2). However, when VSN16R was incubated with mouse, rat, dog, monkey and human hepatocytes, and the supernatants assessed by LC–MS 1 h later, two dominant metabolites from mouse hepatocytes were identified and synthesized (Figure 3A). (R,Z)‐N‐(1‐hydroxypropan‐2‐yl)‐3‐(6‐(methylamino)‐6‐oxohex‐1‐en‐1‐yl)benzamide (molecular weight = 304 Da), named VSN22R, resulted from demethylation of the N‐dimethyl amino group of VSN16R. Oxidation of the terminal alcohol group to form a carboxylic acid produced (Z)‐(3‐(6‐(dimethylamino)‐6‐oxohex‐1‐en‐1‐yl)benzoyl)‐D‐alanine (molecular weight = 332 Da), which was named VSN44R (Figure 3A). VSN22R and VSN44R were tested in the electrically‐evoked contraction assays of the mouse vas deferens, and it was found that VSN22R had comparable affinity (EC_50_ = 9 nM) to VSN16R (EC_50_ = 10 nM), whereas VSN44R was significantly more potent (EC_50_ = 1 nM) (Figure 3B). In contrast, they all had comparable activity (EC_50_ = ~100 nM) in relaxing methoxamine‐evoked contraction of third‐order mesenteric arteries (Figure 3C). However, given the activity in the tissue‐based assays (Figure 3B, C), it was not surprising that both VSN22R and VSN44R could inhibit the spasticity during EAE (Figure 3D). This indicates that they should contribute to the pharmacological effect of VSN16R *in vivo*.

### VSN16R is well tolerated at drug levels achievable in humans

**Figure 4:**
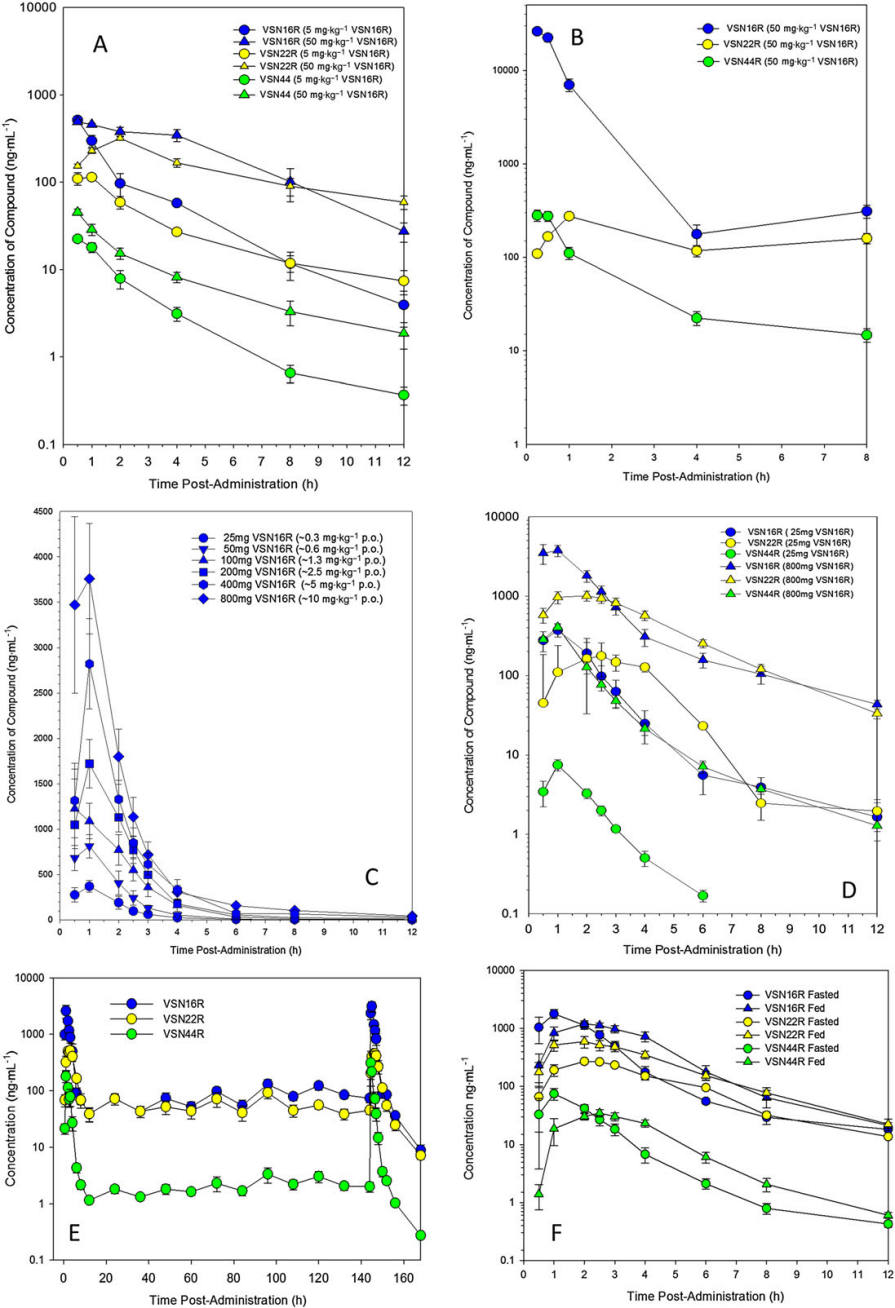
Pharmacokinetics of VSN16R in rats, dogs and humans. Liquid chromatography mass spectrometry plasma levels of VSN16R, VSN22R or VSN44R detected in (A) rat after 5 mg·kg^−1^ or 50 mg·kg^−1^ VSN16R p.o. (*n* = 6 per group) and (B) dogs after 50 mg·kg^−1^ p.o. (*n* = 6). (C) Human VSN16R levels after 25, 50, 100, 200, 400 and 800 mg p.o.; (D) human VSN16R, VSN22R and VSN44R levels after 25 and 800 mg VSN16R p.o. (*n* = 6 subjects per group). (E) Human VSN16R, VSN22R and VSN44R plasma levels after twice daily 400 mg VSN16R p.o., where the 12 h sample was taken immediately before dosing (*n* = 6 humans per group). (F) Human VSN16R, VSN22R and VSN44R plasma levels after 200 mg VSN16R p.o. in people that were fed or fasted before drug administration.

Studies indicated that mice tolerate 1000 mg·kg^−1^ p.o. and likewise, independent, toxicology studies indicated that the no “evidence of adverse events level (NOEL)” was 1000 mg·kg^−1^ p.o. in rats over 28 days (*n* = 20). The NOEL was 150 mg·kg^−1^ p.o. in rabbits and 100 mg·kg^−1^ p.o. in dogs (*n* = 6) with reproductive toxicology safe levels at 1000 mg·kg^−1^ p.o. in rats (*n* = 9) and >500 mg·kg^−1^ in rabbits (*n* = 9), and there was no evidence of mutagenesis in a standard Ames test (Supporting Information Table S3). In rats, there was occasional salivation and ‘ploughing’ behaviours, and there was salivation and vomiting in dogs at doses of 200 mg·kg^−1^ p.o. Likewise, rabbits demonstrated loss of appetite and loss of weight at higher (>150 mg·kg^−1^) doses of VSN16R. These behaviours can be associated with poor taste. This aspect was avoided by encapsulating the drug for human use and no vomiting or issues of taste were reported in human studies (*n* = 60). Histological analysis of animal (>30) tissues showed essentially no significant microscopic findings attributable to VSN16R (Supporting Information Table S4), and there were no cardiovascular issues (Supporting Information Table S5). The drug was therefore very well tolerated in animals. Analysis of plasma levels of a therapeutic 5 mg·kg^−1^ p.o. dose of VSN16R in ABH mice with spastic EAE indicated that there was only 73.0 ± 6.1 ng·mL^−1^ of VSN16R, 106 ± 6.1 ng·mL^−1^ VSN22R and only 2.1 ± 0.3 ng·mL^−1^ VSN44R present (*n* = 4). This plasma level of VSN16R and its metabolites was easily achievable in rats (Figure 4A) and dogs (Figure 4B) in toxicological studies and safety studies in humans (Figure 4C, D). Thirty minutes following 50 mg·kg^−1^ p.o., the amount of VSN16R detected in plasma (Mean ± SD) was 4907 ± 32.3 ng·mL^−1^ (15 μM) in rats (*n* = 6) compared with 26 150 ± 1557 ng·mL^−1^ (82 μM) in dogs (*n* = 6). At C_max_, typically at 30 min following 1000 mg·kg^−1^ p.o., there was up to 117 000 ± 5710 ng·mL^−1^ (368 μM) VSN16R in rats (*n* = 8), and at 200 mg·kg^−1^ p.o., there was 105 283 ± 13 309 ng·mL^−1^ VSN16R in dogs (*n* = 6), and therefore, animals could tolerate high levels of VSN16R.

### VSN16R is well tolerated in humans

Healthy male volunteers 18–50 years old (Supporting Information Table S6) were randomly allocated to treatment in double‐blind, placebo‐controlled, phase I studies (EudraCT 2013‐002765‐18). This consisted of a single ascending dose (SAD) study of 25, 50, 100, 200, 400 and 800 mg in groups of eight different people per dose with six people given active drug in either 25 or 100 mg gelatine capsules (Figure 4C). No serious adverse drug‐related events occurred, and treatment was largely unremarkable in all cases. Any adverse events reported (14.3% in SAD) were mild, and none were considered to be definitively related to VSN16R, although no adverse event was recorded in people treated with placebo capsules. However, 5/42 VSN16R‐treated subjects (11.9%) were considered to have a possible associated effect with treatment; these included dizziness (one subject at 25 and 50 mg), headache (one subject at 200 mg) and dyspepsia and nausea (at 800 mg). Haematology and serum biochemistry (Supporting Information Table S7), coagulation, urine analysis, vital signs and electrocardiograms (Supporting Information Table S8) and blood pressure (10 min in a supine position or after 1 min standing; Supporting Information Table S9) remained unremarkable. Plasma was repeatedly sampled, and pharmacokinetic studies demonstrated the rapid presence of VSN16R indicating good gut absorption, with 100% bioavailability at 25 mg and a terminal elimination of 3.4–4.9 h over the dose range studied (Figure 4C). Even at the lowest drug dose tested (25 mg), it was clear that plasma levels were well above plasma levels of therapeutic 5 mg·kg^−1^ p.o. doses in mice, and these were achieved for a number of hours (Figure 4C, D). Formation of metabolites was rapid, and it was found that VSN22R was the dominant metabolite species in humans and once generated levels were similar to those found with VSN16R and had a terminal elimination half‐life of 4.0–5.3 h across the dose range tested (Figure 4A–C). Again VSN44R appeared at 10‐ to 100‐fold lower levels (Figure 4A–C). These data suggest that even in the absence of a slow‐release formulation, twice (b.i.d)‐thrice daily drug delivery could probably achieve steady state, therapeutic drug levels (Figure 4F). In addition to the trial containing a multiple ascending drug dose study of twice daily 25, 100 and 400 mg in groups of eight different fasted people per dose, six people were given the active drug at doses of either 25 or 100 mg in gelatine capsules (Figure 4D). Again, the drug was well tolerated and the pharmacokinetics resembled that following the SAD studies (Figure 4C–E). It was found that 6/18 VSN16R‐treated subjects (33.3%) and to 2/6 (33.3%) placebo subjects reported adverse events and 3/18 VSN16R‐treated subjects (16.7%) were considered to have a possible side effect of the treatment, although again none was considered to be definitely related to drug treatment. These people reported oropharngeal pain and rhinitis (at 25 mg b.i.d), abdominal distension (at 25 mg b.i.d) and abdominal pain and nausea (at 400 mg b.i.d). There were no serious adverse events. Following twice daily dosing, steady‐state plasma levels above 100 nM for both VSN16R and VSN22R, likely to be therapeutic, could be achieved in healthy individuals (Figure 4D). Initial studies were in fasted individuals (Figure 4C–E), and therefore, studies were undertaken following feeding with 0.5 h of dosing. Whilst this delayed the time of the C_max_ (T_max_) and reduced C_max_, the extent of absorption (total exposures) was not influenced and if anything, feeding improved the therapeutic levels of VSN16R and its metabolites (Figure 4F). These data indicate that VSN16R is well tolerated in humans and does not induce sedation.

### VSN16R targets BK_Ca_ potassium channels independently of G‐protein‐coupled cannabinoid receptors

**Figure 5:**
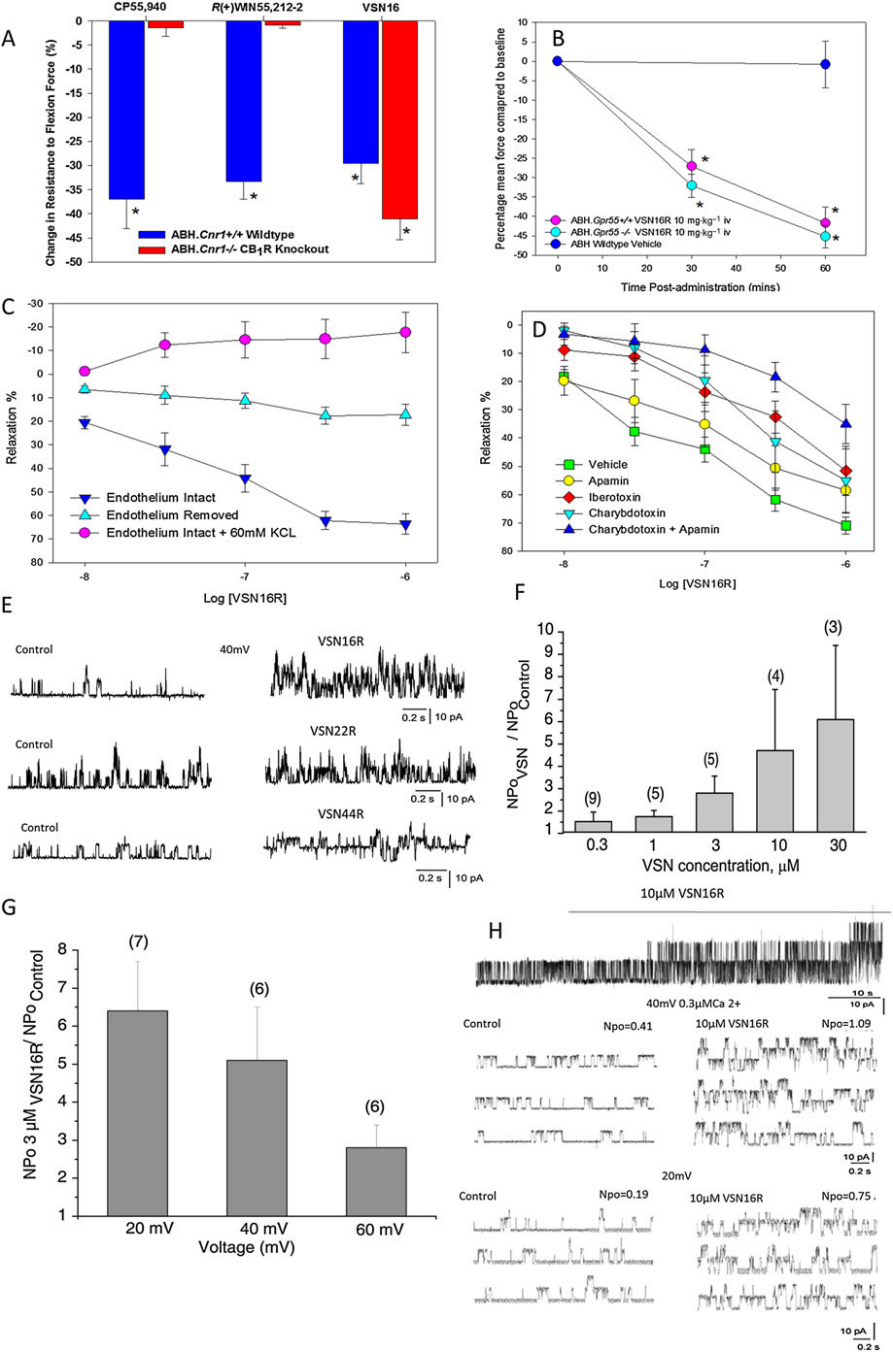
VSN16R is a BK_Ca_ channel opener. (A) Percentage change in resistance to flexion in response to treatment with either 1 mg·kg^−1^ i.p. CP55,950 (*n* = 5 animals, *n* = 8 limbs), 5 mg·kg^−1^ i.p. R(+)WIN55,212 (*n* = 7 animals, *n* = 14 limbs) or 5 mg·kg^−1^ i.v. VSN16R in wild‐type ABH (*n* = 7 animals, *n* = 14 limbs) or congenic CB_1_ receptor‐deficient mice (*n* = 5 animals per group *n* = 10 limbs) at 30 min after treatment. (B) Changes in resistance to flexion in response to treatment with vehicle (*n* = 8 animals, *n* = 14 limbs) or 10 mg·kg^−1^ i.v. VSN16R wild‐type ABH (*n* = 8 animals, *n* = 16 limbs) or congenic GPR55‐deficient mice (*n* = 8 animals, *n* = 15 limbs). (C, D) Dose‐dependent VSN16R‐mediated relaxation of methoxamine‐evoked contractions in rat mesenteric arteries. (C) Endothelial intact (*n* = 11 assays), endothelium denuded (*n* = 6 assays) or endothelium intact cultures in the presence of 60 mM KCl (*n* = 4 assays). (D) Vehicle‐treated controls (*n* = 17 assays) or pretreated with 50 nM apamin (*n* = 7 assays), 50 nM iberotoxin (*n* = 5 assays), 50 nM charybdotoxin (*n* = 6 assays) or a combination of apamin and charybdotoxin (*n* = 6 assays). (E) At 3 μM VSN16R, VSN22R and VSN44R stimulate single BK_Ca_ channel activity in inside‐out patches, excised from EA.hy926 cells, held at 40 mV and exposed to 0.3 μM free Ca^2+^; representative plots are shown. (F, G) Statistical representation of the potentiation of BK_Ca_ single channel activity (*N*Po) by 3 μM VSN16R. (F) Concentration‐dependent increase in *N*Po by 0.3–30 μM VSN16R at a holding voltage of 60 mV and (G) different voltages and fixed Ca^2+^ 300 nM. The number of patches is shown in brackets. The results represent the mean ± SD. (H) VSN16R stimulates single BK_Ca_ channel activity in inside‐out patch excised from human neural HCN‐2 cells and held at either 20 or 40 mV in the presence of 0.3 μM free Ca^2+^ (*n* = 4 patches).

VSN16R was shown to be a safe and potent anti‐spastic agent, but its mechanism of action was not initially obvious, as early in the studies it was recognized that, despite being antagonized by SR141716A (Figure 1A), it does not bind to or agonize CB_1_ receptors in receptor‐transfected cell line‐based assays (Supporting Information Figure S5). Furthermore, VSN16 could still significantly inhibit spasticity in global CB_1_ receptor‐deficient ABH mice (Figure 5A). Therefore, the action of VSN16R was clearly independent of CB_1_ receptors. In addition, VSN16R did not bind (tested to 10 μM) to CB_2_ receptors or other parts of the endocannabinoid and endovanilloid system (tested to 10 μM) and a wide range of receptors and transporters (Supporting Information Figure S5).

Although SR141716A and AM251 are CB_1_ receptor antagonists, they have additional off‐target effects associated with a non CB_1_/CB_2_ receptor vascular target of anandamide (Ho and Randall, 2007; Pertwee *et al.,* 2010). These compounds and O‐1918 antagonize the action of VSN16R in mesenteric artery relaxation assays (Hoi *et al.,* 2007), and these effects were used to help identify the target for VSN16R activity. It has been reported that O‐1918 can target **GPR18** and GPR55, candidates for a putative non‐CB_1_/CB_2_ vascular cannabinoid receptor that is activated by some endocannabinoids (Baker *et al.,* 2006; McHugh *et al.,* 2010; Bondarenko, 2014). Stimulation of this vascular target has been associated with the development of low blood pressure in some studies (Bondarenko, 2014); however, it was clear that VSN16R did not induce hypotension in rodents (Hoi *et al.,* 2007), dogs and humans (Supporting Information Tables 5S and S9). GPR18 and notably GPR55 are bound by SR141716A and AM251 (Baker *et al.,* 2006; Pertwee *et al.,* 2010). However, as the action of VSN16R is *Bordetella pertussis* toxin‐insensitive, the vasorelaxing effect of VSN16R is not mediated by GPR18 (Hoi *et al.,* 2007). Furthermore, VSN16R does not directly bind to GPR55 either [tested in Multispan C1113, Multispan H113, HEK293T.*GPR55* cells lines and mouse DBT.*Gpr55* cell lines in a variety of different assays including calcium signalling, receptor internalization and nuclear localization of cAMP response element binding protein (Supporting Information Figure S5)]. Importantly, VSN16R continued to inhibit spasticity in GPR55‐deficient ABH mice (Figure 5B). Therefore, other O‐1918 reactive targets were examined.

Although VSN16 was originally designed as an anandamide analogue, it is also structurally similar to N‐arachidonoyl glycine and N‐arachidonoyl serine (Supporting Information Figure S1). Arachidonoyl glycine is an endogenous ligand of GPR18 that causes vasorelaxation, which is blocked by O‐1918, *via* stimulation of a presumed G‐protein‐coupled vascular receptor (Parmar and Ho, 2010). However, this appears to directly activate big conductance calcium‐activated potassium (BK_Ca_) channels (Bondarenko *et al.,* 2013). Likewise, N‐arachidonoyl serine that may act on GPR55 can inhibit vasorelaxation by endothelial cell‐dependent and independent mechanisms following direct BK_Ca_ activity that was directly blocked by O‐1918 (Godlewski *et al.,* 2009). This suggested that BK_Ca_ channels may mediate the vasorelaxing effects of at least some cannabinoids (Bondarenko, 2014). VSN16R had no effects on the primary porcine aorta endothelium (Supporting Information Figure S4), but in assays in third‐order rat mesenteric arteries, VSN16R induce significant vasorelaxation in an endothelium‐dependent manner (Figure 5C) and this effect was significantly inhibited by antagonists of BK_Ca_ channels, notably by iberotoxin and charybdotoxin. Apamin, a K_Ca_2.2/2.3 (*KCNN2*/*KCNN3*) channel antagonist, alone had a small inhibitory effect on VSN16R function in the mesenteric artery assay (Figure 5D), but VSN16R failed to block the binding of apamin to K_Ca_2.2 channels in transfected cells (Supporting Information Figure S5). In addition, the relaxation was dependent on potassium flux, as VSN16R produced no relaxation in the presence of extracellular 60 mM KCl, supporting an action *via* BK_Ca_ channels (Figure 5C).

**Figure 6:**
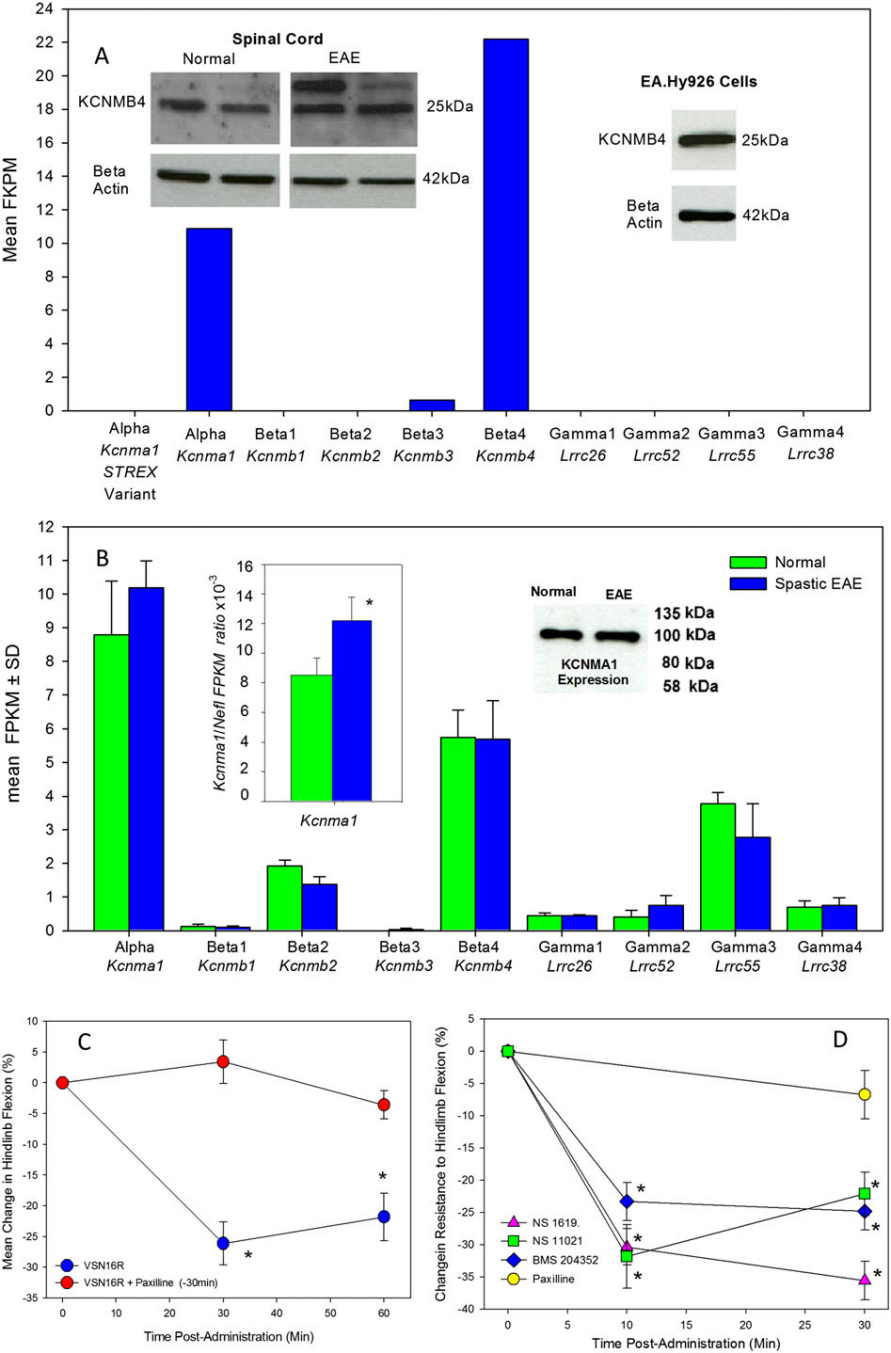
BK_Ca_ openers control spasticity. (A) RNAseq expression of BK_Ca_ channel components in EA.hy926 cells. Inset represents the western blot of KCNMB4 in EA.hy926 cells, which was tested in four samples, and western blot of KCNMB4 expression in the spinal cord in control (*n* = 3) and spastic EAE spinal cord tissues (two shown, *n* = 6 total) showing an additional prominent band, not present in EA.hy926 cells, above the anticipated fragment size more notable during EAE. This antibody detected multiple bands in western blot analyses. (B) RNAseq expression of BK_Ca_ channel components in the spinal cords of spastic EAE (*n* = 3 mice) and age‐matched normal mice (*n* = 3 mice). The results represent the mean ± SD. Inset represents the western blot of KCNMA1 in control and spastic EAE tissue (Antibody APC‐021 detected a single band at the anticipated size and was repeated with antibody APC‐151 with comparable results), and insets show a significant change in ratio between *Kcnma1* and neurofilament light (*Nefl*) gene levels. (C) Percentage change in resistance to flexion of spastic hindlimbs in ABH mice in response to treatment with 5 mg·kg^−1^ p.o. VSN16R in water (*n* = 10 animals, *n* = 19 limbs) that were pretreated with saline or 1 mg·kg^−1^ i.p. paxilline (*n* = 7 animals, *n* = 14 limbs). (D) Percentage change in resistance to flexion of hindlimbs in ABH mice following injection i.p. with 1 mg·kg^−1^ paxilline (*n* = 7 animals, *n* = 13 limbs), 40 mg·kg^−1^ i.p. BMS‐204352 (*n* = 7 animals, *n* = 13 limbs), 20 mg·kg^−1^ i.p. NS‐1619 (*n* = 7 animals, *n* = 14 limbs) or 10 mg·kg^−1^ i.p. NS‐11021 (*n* = 7 animals, *n* = 13 limbs). *Significant difference compared with baseline.

This target was definitively shown in single channel, inside‐out patch clamp experiments in human EA.hy926 cells (Bondarenko *et al.,* 2011), where VSN16R (*n* = 17 patches), VSN22R (*n* = 22 patches) and VSN44R (*n* = 4 patches) all facilitated single BK_Ca_ channel activity when applied to the inner surface of the membrane (Figure 5E). The increase in *N*Po occurred in a concentration‐, calcium‐ and voltage‐dependent manner (Figures 5F, G; Supporting Information Figure S3), indicating that the compounds directly act as BK_Ca_ channel openers, without binding to other receptors; this causes hyperpolarization of the membrane. Importantly, this mechanism was active in human HCN‐2 neural cells where VSN16R stimulated single BK_Ca_ channel activity in inside‐out patches (Figure 5H), providing further evidence that VSN16R acts directly *via* neuronal BK_Ca_ channels (Figure 5H). Consistent with this, RNA*seq* indicated that EA.hy926 cells essentially only express the neural, KCNMA1, KCNMB4 BK_Ca_ isoform (Figure 6A). They did not express any of the leucine‐rich repeats containing BK_Ca_ γ subunit proteins (Zhang and Yan, 2014) or the STREX (Xie and McCobb, 1998) steroid‐responsive KCNMA1 splice variant (Figure 6A).

### BK_Ca_ channels are dysregulated during EAE and are a novel mechanism to control spasticity

In the spinal cord, the dominant BK_Ca_ subunits are the KCNMA1 α pore and the KCNMB4 β chain, and KCNMB2 to a lesser extent and the LRRC52 γ chain (Figure 6B). Surprisingly, *RNAseq* analysis of spinal cords from spastic and age‐matched controls (*n* = 3) failed to detect significant differences in the expression of any of the BK_Ca_ subunits (Figure 6B), consistent with KCNMA1‐specific western blot of spinal cords (Figure 6B). Likewise, western blot of KCNMB4 in spinal cords did not indicate differences of KCNMB4 levels in spastic EAE, although additional bands, not present in EAE.hy926, became prominent in spastic mice with EAE (*n* = 6) compared with controls (*n* = 3), possibly due to disease‐related post‐translational modifications (Figure 6A), such as glycosylation (Jin *et al.,* 2002). However, as spastic EAE is associated with significant nerve loss (Pryce *et al.,* 2003), when KCNMA1 expression was normalized to neurofilament content, there was a significant increase in the KCNMA1 (*Kcnma1*) to neurofilament light (*Nefl*) ratio in spastic mice (Figure 6B), suggesting some dysregulation during the disease. However, blockade of the receptor with paxilline did not influence the baseline level of spasticity (Figure 6C, D). Animals with a deficiency in BK_Ca_ channels can exhibit adverse motor phenotypes and abnormal development that prelude their use in spasticity studies (Brenner *et al.,* 2000; Sausbier *et al.,* 2004; Martinez‐Espinosa *et al.,* 2014); therefore, a pharmacological approach was used to investigate the involvement of BK_Ca_ channels in the effects of VSN16R (Figure 6C). It was found that 1 mg·kg^−1^ i.p. of the BK_Ca_ antagonist paxilline, which is just below a tremorogenic dose in mice (Imlach *et al.,* 2008), could completely inhibit the action of VSN16R (Figure 6C). Furthermore, when structurally unrelated BK_Ca_ openers were investigated in spasticity in EAE, it was found that BMS‐204352, NS‐1619 and NS‐11021, used at published *in vivo* i.p. doses, could all significantly inhibit spasticity (Figure 6D), without causing sedation. These findings confirm that BK_Ca_ potassium channel openers could provide a novel mechanism to control spasticity.

## Discussion

This study demonstrates that neural BK_Ca_ channels are a previously unrecognized target for control of spasticity in MS and other conditions. As such, compounds that support the opening of the BK_Ca_ channels and hyperpolarize neural membranes will limit neural excitability to control spasticity, in the absence of sedating side effects. These could also potentially affect and may be of value in the control of other MS symptoms or other conditions associated with neural hyperactivity and BK_Ca_ mutations: such as epilepsy, neuropathic pain, tinnitus and fragile X syndrome (Sausbier *et al.,* 2004; Brenner *et al.,* 2005; N'Gouemo, 2014; Liu *et al.,* 2015). The opening of calcium‐activated potassium channels forms part of the neuronal repolarizing mechanism that helps reset the ionic balance of the nerve to facilitate the generation of further action potentials (Sausbier *et al.,* 2004; Brenner *et al.,* 2005; N'Gouemo, 2014). The observed up‐regulation of these channels in spinal cords probably contributes to facilitating the overexcitation of motor nerves leading to spasticity. As NS‐1619, which is a KCNMA1‐selective agent (Gessner *et al.,* 2012), can control spasticity, this indicates that potassium‐induced neural membrane hyperpolarization following the opening of the α pore in BK_Ca_ channels is the important mechanism of action of this compound. VSN16R also induces a similar effect by this mechanism, which will limit the generation of action potentials and control spasticity.

Although VSN16R and NS‐1619 both maintain the opening of BK_Ca_ channels, *via* the KCNMA1 pore, to produce membrane currents and both have their effects blocked by the actions of iberotoxin and SR141617A (White and Hiley, 1998), they have subtly distinct pharmacology and molecular targets (White and Hiley, 1998; Begg *et al.,* 2003; Hoi *et al.,* 2007; Gessner *et al.,* 2012). NS‐1619 opens BK_Ca_ *via* a direct effect on the KCNMA1 pore of the channel and does not require the presence of KCNMB β chains for activity (White and Hiley, 1998; Gessner *et al.,* 2012). Furthermore, in contrast to VSN16R, the action of NS‐1619 is resistant to the antagonistic effect of O‐1918 (Gessner *et al.,* 2012). As such, NS‐1619 relaxes mesenteric arteries *via* an additional, endothelial‐independent mechanism that is due to an action on the KCNMA1 pore in smooth muscle that express the KCNMA1, KCNMB1 BK_Ca_ isoform (White and Hiley, 1998; Brenner *et al.,* 2000; Papassotiriou *et al.,* 2000; Sausbier *et al.,* 2004). VSN16R does not activate KCNMA1 directly or act *via* the smooth muscle BK_Ca_ isoform, as we showed here that its ability to induce arterial relaxation is endothelial cell‐dependent. Also VSN16R does not induce relaxation of porcine aorta that express low levels of KCNMA1 and no BK_Ca_ β chains (Papassotiriou *et al.,* 2000) and the lacks an effect on βγ‐methylene ATP activity in smooth muscle. This is consistent with the lack of influence of VSN16R on blood pressure observed in rats, dogs and humans, which is controlled by KCNMA1 and KCNMB1 on smooth muscle (Brenner *et al.,* 2000; Sausbier *et al.,* 2004; Zheng *et al.,* 2013). Likewise, VSN16R does not need the presence of BK_Ca_ γ chains for activity. Therefore, although the effects of VSN16R are associated with membrane hyperpolarization that results from KCNMA1 pore opening, maintaining the opening of the pore must occur *via* interactions of other elements (KCNMB4) of the BK_Ca_ channels. Skeletal muscles express only trace levels of BK_Ca_ (KNCMA1) and do not appear to express KCNMB1‐4 (Freeman *et al.,* 1998; Bednarczyk *et al.,* 2013) and are therefore unlikely to be responsive to VSN16R. However, a neuronal action of VSN16R was indicated *via* the electrophysiological control of spasticity and the finding that a VSN16R‐responsive cell line essentially only expresses a single KCNMA/KCNMB4 BK_Ca_ channel, which is the major neuronal BK_Ca_ isotype (Bednarczyk *et al.,* 2013). Neuronal BK_Ca_ channels are found at high levels within sensory nerves, notably within dorsal root ganglia and spinal nerves (Allen Spinal Cord Atlas, 2008; Freeman *et al.,* 1998), where they may affect motor nerves or inhibitory interneurons, with or without influences on sensory outputs, as has been found with cannabinoid receptor agonists (Pryce *et al.,* 2014).

Further work is required to determine the precise location and molecular function of VSN16R on BK_Ca_ channels during spasticity. However, this is complicated, as BK_Ca_ gene knockout animals exhibit adverse neurological phenotypes including premature death, ataxia, tremors and gait and movement problems, which preclude their use in spasticity *in vivo* (Brenner *et al.,* 2000; Sausbier *et al.,* 2004).Nevertheless, this may highlight new indications for treatment with VSN16R or related molecules. Furthermore, the structural and electrophysiological biology of BK_Ca_ channels is highly complex because of their multimeric structure of alternatively spliced and post‐translationally modified α, β and γ chains, which modulate both the expression of the α chain and the calcium and voltage thresholds of the α pore in both positive and negative ways (Brenner *et al.,* 2000; Weiger *et al.,* 2000; Sausbier *et al.,* 2004; Zhang and Yan, 2014). As such, both gain and loss of function of the BK_Ca_ channels can lead to neural hyperexcitability (N'Gouemo, 2014). The BK_Ca_ channels also appear to be part of the signalling machinery of some cannabinoid‐related GPCRs, such as GPR55 (Bondarenko *et al.,* 2011; Bondarenko, 2014) and potentially the CB_1_ receptor (Sánchez‐Pastor *et al.,* 2014). In addition, being calcium‐dependent, BK_Ca_ channel activity and VSN16R function may also be modulated secondary to alterations to intracellular calcium stimulation, or other intracellular signalling molecules, following activity of other receptors. This may be a mechanism by which SR141617A can inhibit BK_Ca_ activity in tissue assays (White and Hiley, 1998). This type of activity may contribute to the confusion around the pharmacology of endocannabinoids (Howlett *et al.,* 2002; Pertwee *et al.,* 2010; Bondarenko, 2014). Additional actions of VSN16R cannot be excluded, especially since anandamide has a number of targets (Pertwee *et al.,* 2010), but a large number of relevant receptors and channels were excluded as potential targets. Importantly, the pharmacological action of VSN16R was clearly blocked by antagonists of the BK_Ca_ channel and demonstrates that this is a new target to control spasticity.

This study indicates that potassium‐induced hyperpolarization following opening of the α pore in BK_Ca_ channels is a novel central mechanism of action of these anti‐spastic drugs and, in neural membranes, will act to limit excessive action potential generation. Such membrane hyperpolarization is the common mechanism of action of many anti‐spastic agents, which counter excitatory depolarizing signals that can lead to spasticity (Toda *et al.,* 2014; Trompetto *et al.,* 2014). This pathway is mediated directly through the opening of BK_Ca_ potassium channels, as shown here, and *via* calcium channel inhibition and notably activation of inwardly rectifying potassium channels after G protein signalling with CB_1_ receptor and GABA_B_ agonists, and through chloride ion influx following stimulation of GABA_A_ receptors (Isomoto *et al.,* 1997; Howlett *et al.,* 2002; Pertwee *et al.,* 2010). However, in contrast to cannabinoids and GABA agonists, VSN16R is well tolerated in animals, even following direct injection into the brain. Likewise, BMS‐204352, which is highly hydrophobic and readily enters the brain, also has a large therapeutic window in rodents (Gribkoff *et al.,* 2001; Kristensen *et al.,* 2011). As BK_Ca_ channels are activated in depolarizing conditions, it is possible that the BK_Ca_ channel conformation targeted by VSN16R may only be active in certain states. As such, it is of interest that NS‐1619 fails to influence acute nociception but controls chronic neuropathic pain (Chen *et al.,* 2009), suggesting that the certain types of BK_Ca_ channel may be pathologically expressed. This may contribute to the lack of sedation observed here in both animals and humans. As the first example of a neuronal‐selective BK_Ca_ channel ligand, VSN16R may be the prototype for a new range of well‐tolerated drugs with applications in diverse conditions in which excitotoxicity is evident or where the channel has a specific role. VSN16R was originally designed as a cyclic analogue of anandamide to exploit the benefit that the endocannabinoid system has to offer. Importantly, however, it moves us away from the cannabis plant, and all its issues of recreational use, towards a novel pharmaceutical class of agents that offers therapeutic benefits without the psychoactive effects associated with cannabis use (Howlett *et al.,* 2002; Varvel *et al.,* 2005), or the sedating effects of other current anti‐spastic agents (Shakespeare *et al.,* 2003). This suggests that VSN16R is ready for testing in people with spasticity in MS (EudraCT 2014‐004412‐11, NCT02542787) as a treatment for this neurological disease.

## Author contributions

D.B., G.P. and D.L.S. conceptualized the study. C.V., M.O. and D.L.S were responsible for chemistry and compound design. A.I.B., D.B., D.L.S., G.P., M.D.B., G.G., J.S., W.S.V.H. and R.G.P. did the experimental design. Tissue‐based *in vitro* assays were conducted by A.I.B., A.J.I., C.M.H., C.T., I.S., L.S., R.R., S.S., A.J.I. and W.S.V.H. Production and management of mouse lines were done by D.B., G.P., S.S. and S.J.J.; *in vivo* assays were done by D.B., G.P., M.D.B., S.A., S.S. and T.E.W. Phase I Trial development was performed by J.S., K.P. and G.G.; A.I.B., D.B., D.L.S., C.V., G.G., J.S., K.P., S.J.J. and W.F.G. were responsible for the funding of the study, and all authors contributed to the final manuscript.

## Conflict of interest

Some of the authors (D.B., C.V., G.P. and D.L.S.) have filed patents based on the work within this study. D.B., C.V. and D.L.S. are founders and shareholders of Canbex Therapeutics Limited, a UCL spin‐out company aiming to develop VSN16‐related compounds. G.P. and G.G. are shareholders of Canbex Therapeutics. J.S. and K.P. manage and are shareholders of Canbex Therapeutics, and D.B., G.G. and D.L.S. are consultants to Canbex Therapeutics. The Institutions of A.I.B., A.J.I., D.B., D.L.S., R.G.P., R.R., W.F.G. and W.S.V.H. received funds from Canbex Therapeutics to support VSN16R‐related research.

## Declaration of transparency and scientific rigour

This Declaration acknowledges that this paper adheres to the principles for transparent reporting and scientific rigour of preclinical research recommended by funding agencies, publishers and other organisations engaged with supporting research.

## Supporting information


**Figure S1** *Biological conformations of the arachidonic acid chain*. (**A, B**) “C” type conformations of the polyene chain from diverse biological targets from X‐ray. This suggested that conformational restriction of (**C**) anandamide would be useful approach to generate novel molecules as in (**D**) VSN16. This has structural similarities with (**E**) N‐arachidonoyl glycine and (**F**) N‐arachidonoyl serine.
**Figure S2** *VSN16R does not inhibit β γ methylene adenosine triphosphatase‐induced muscle contraction in the vas deferens.* Mouse vas deferens were treated with either DMSO vehicle or 100 nM VSN16R 30 min before the first organ bath injection of various concentrations of βγ‐methylene ATP into the organ bath . The results represent the mean ± SEM of βγ‐methylene ATP‐induced increases in tension (expressed in grams) of electrically unstimulated vasa deferentia. (*n* = 6/group).
**Figure S3** *Anaesthetics inhibit spasticity and muscle tone and can interfere with the action of VSN16R*. (**A**) Strain gauge recording before and after (5 min) the induction of ketamine and medetomide anaesthesia, typically used for rodent electrophysiology studies. (**B**) Loss of muscle tone of spastic animals following anaesthetic showing measurement of resistance to limb flexion before and 5 min after anaesthesia.*** significant compared to baseline before anaesthetic using paired *t*‐test (*n* = 5 animals) (**C**) The magnitude of the H‐wave was measured in the shin muscle in spastic animals following sciatic nerve stimulation (100% = H wave at administration of 30 mg/kg i.v. VSN16R in PBS at 0 min). Although *n* = 0/3 PBS‐treated animals showed an inhibition of the H reflex, this could be inhibited by VSN16R (**D**) Electrophysiological trace of the shin muscle following stimulation of a spastic mouse before and after 30 mg/kg i.v. VSN16R administration. (**E**) However, the H reflex was only inhibited in some (3/5) animals responded (Green circles), whereas others did not (blue circles). Trace of individual mice (**F**) However, it was subsequently found that ketamine (200 μM. To represent anaesthetic levels in blood) can inhibit the mechanism of action of VSN16R in part of the dose–response curve to methoxamine‐evoked contraction of rat mesenteric artery (*n* = 5–6 cultures). Ketamine blocks NMDA receptors, that limits calcium ion fluxes that can influence BK_Ca_ function and can inhibit the inside‐out current of BKCa channels with an EC_50_ = ~25 μM (Denson DD *et al.* Brain Res 638:61). In contrast to healthy animals, mice with spasticity did not tolerate anaesthetics, which caused death in some instances, prompting discontinuation of the approach prior to attempted dose reduction.
**Figure S4** *Patch Clamp analysis of VSN16R activity on BK*_*Ca*_ *channels*. (**A**) Primary pig aorta do not respond to VSN16R (*n* = 3 patches). Time course of the current development at −100 mV (lower) and +85 mV (upper) in response to VSN16R or the removal of potassium. (**B**) Whole cell currents of human EA.hy926 cells in response to voltage ramps before (control), and during exposure to 15 μM VSN16R in the absence and presence of 2 μM paxilline. The current represents the influence of endogenously expressed conducting ion channels within the cell, but the sensitivity to paxilline indicates that the majority response was mediated by BKca channels. (**C‐E**) This was shown in single channel patch clamp experiments in EA.hy926 cells. (**C**) Voltage dependence of activity of VSN16R in inside‐out patch clamp of single BK_Ca_ channels VSN16R. (**D**) Calcium dependence of activity of VSN16R. Single BK_Ca_ channel activity in inside‐out patch held at +60 mV and exposed to either 1 μM or 0.3 μM free Ca^2+^ concentrations before (control) and after treatment with 3 μM VSN16R. (**E**) VSN16R exhibits a concentration‐dependent induction of potassium currents in inside‐out patch clamp of single BK_Ca_ in EA.hy926 cells. The patch was held at 20 mV and exposed to 0.3 μM free Ca^2+^ under symmetrical K+ conditions. Representative traces that were repeated.
**Figure S5** *Receptors and other targets that VSN16R fails to bind/activate* (**A**) Receptors lacking activity with 10 μM VSN16R. Binding assays and positive controls were performed by Cerep, Multispan, DiscoverX, MDS pharma and Chantest. (**B**) Lack of activity of VSN16R on CB_1_ and CB_2_ cannabinoid receptors (**C**) Relative lack of activity of VSN16R agonism on U20S cells transfected with human GPR119 compared with oleoylethanolamide as assessed using cyclic AMP assay (**D**). HEK293 cells do not respond to lysophosphoinositol (LPI. GPR55 agonist) stimulation, but respond to lysophophatidic acid (LPA), unless they are transfected with human *GPR55* (left) as assessed using calcium ion fluxes (Henstridge CM *et al.* Br J Pharmacol 2010; 160:604). (**E**) HEK293.*GPR55* demonstrate calcium fluxes following stimulation with AM251 but do not respond to VSN16R (**F**) Lack of activity of VSN16R on DBT cells stably transfected with mouse *Gpr55*. These were incubated with 1‐3 μM LPI, 10 μM VSN16A or the 10 μM and the nuclear expression of cAMP response element‐binding protein (CREB) was assessed by immunocytochemistry (Henstridge *et al.* 2010).
**Table S1** *VSN16R does not induce neurobehavioural behavioural tests in an Irwin test.*
**Table S2** *VSN16R does not induce cytochrome P450 enzymes.*
**Table S3** *VSN16R does not induce chromosomal mutagenesis.*
**Table S4** *VSN16R does not induce tissue toxicology of VSN16R in rats and dogs.*
**Table S5** *Lack of hypotension induced by VSN16R in dogs.*
**Table S6** *Demographics of humans in phase I double‐blind, placebo‐controlled trial.*
**Table S7** *Single Dose of VSN16R did not affect haematology and blood chemistry in humans.*
**Table S8** *Single dose of VSN16R did not affect coagulation, urinanalysis, vital signs and electrocardiograms in humans.*
**Table S9** *Lack of hypotension induced by VSN16R in humans.*
