# Supplementary material for: Big conductance calcium‐activated potassium channel openers control spasticity without sedation
Source: Br J Pharmacol. 2017 Jul 7;174(16):2662–81. doi: 10.1111/bph.13889 (PMC5522996; doi:10.1111/bph.13889)
Supplement: Supplementary file 1 — Figure S1 Biological conformations of the arachidonic acid chain. (A, B) “C” type conformations of the polyene chain from diverse biological targets from X‐ray. This suggested that conformational restriction of (C) anandamide would be useful approach to generate novel molecules as in (D) VSN16. This has structural similarities with (E) N‐arachidonoyl glycine and (F) N‐arachidonoyl serine. Figure S2 VSN16R does not inhibit β γ methylene adenosine triphosphatase‐induced muscle contraction in the vas deferens. Mouse vas deferens were treated with either DMSO vehicle or 100 nM VSN16R 30 min before the first organ bath injection of various concentrations of βγ‐methylene ATP into the organ bath . The results represent the mean ± SEM of βγ‐methylene ATP‐induced increases in tension (expressed in grams) of electrically unstimulated vasa deferentia. (n = 6/group). Figure S3 Anaesthetics inhibit spasticity and muscle tone and can interfere with the action of VSN16R. (A) Strain gauge recording before and after (5 min) the induction of ketamine and medetomide anaesthesia, typically used for rodent electrophysiology studies. (B) Loss of muscle tone of spastic animals following anaesthetic showing measurement of resistance to limb flexion before and 5 min after anaesthesia.*** significant compared to baseline before anaesthetic using paired t‐test (n = 5 animals) (C) The magnitude of the H‐wave was measured in the shin muscle in spastic animals following sciatic nerve stimulation (100% = H wave at administration of 30 mg/kg i.v. VSN16R in PBS at 0 min). Although n = 0/3 PBS‐treated animals showed an inhibition of the H reflex, this could be inhibited by VSN16R (D) Electrophysiological trace of the shin muscle following stimulation of a spastic mouse before and after 30 mg/kg i.v. VSN16R administration. (E) However, the H reflex was only inhibited in some (3/5) animals responded (Green circles), whereas others did not (blue circles). Trace of individual mice (F) However, it was su [file BPH-174-2662-s001.pdf]

SUPPORTING INFORMATION

Baker D et al., Big conductance calcium-activatted potassium channel openers control spasticity without sedation. https:/doi.org/10.1111/bph.13889

METHODS 1S. VSN44 preparation

Methyl (Z)-(3-(6-(dimethylamino)-6-oxohex-1-en-1-yl)benzoyl)-D-alaninate

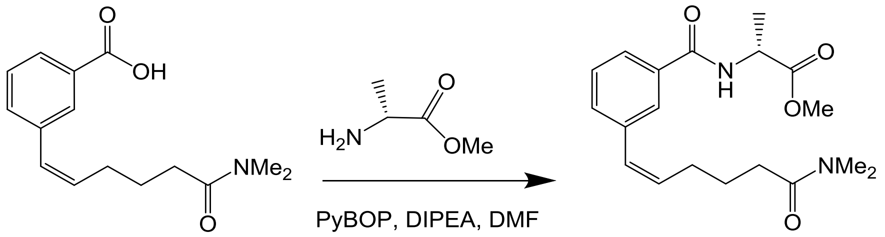

methyl (*Z*)-(3-(6-(dimethylamino)-6-oxohex-1-en-1-yl)benzoyl)-*D*-alaninate

To the substituted benzoic acid (139 mg, 1 mmol) in DMF (1 mL) was added the Ala(OMe) in DMF (1 mL) and the PyBOP (572 mg, 1.1 mmol) added in DMF (2 mL). DIPEA (142 mg, 191 µL, 1.1 mmol) was added dropwise, and the reaction stirred at room temperature overnight. Water (50 mL) was added and ethyl acetate (100 mL). The layers were stirred (5 mins), separated, and the ethyl acetate layer washed with brine (2 x 100 mL), dried (Na_2SO_4) to give the crude product (650 mg). This was flash chromatographed using a 25g Puriflash (silica) column, cyclohexane: acetone 15-45% gradient. Yield 180 mg, 0.54 mmol, 54%.

^{1}H NMR (500 MHz, $CDCl_3$) δ 7.77 (s, 1H), 7.71 (dt, J = 1.6, 7.4, 1H), 7.42 – 7.38 (m, J = 7.4, 1H), 7.38 – 7.31 (m, 2H), 6.46 (d, J = 11.6, 1H), 5.74 (dt, J = 7.7, 11.6, 1H), 4.84 – 4.76 (m, J = 7.2, 1H), 3.77 (s, 3H), 2.95 (s, 3H), 2.90 (s, 3H), 2.42 – 2.30 (m, 4H), 1.83 (p, J = 7.2, 2H), 1.64 (s, 2H), 1.54 (d, J = 7.2, 3H).

^{13}C NMR (126 MHz, $CDCl_3$) δ 173.78, 172.63, 167.13, 137.88, 134.10, 133.23, 132.09, 128.90, 128.53, 127.12, 125.77, 52.54, 48.67, 37.30, 35.54, 32.60, 28.30, 24.99, 18.31, 17.66

(Z)-(3-(6-(dimethylamino)-6-oxohex-1-en-1-yl)benzoyl)-D-alanine

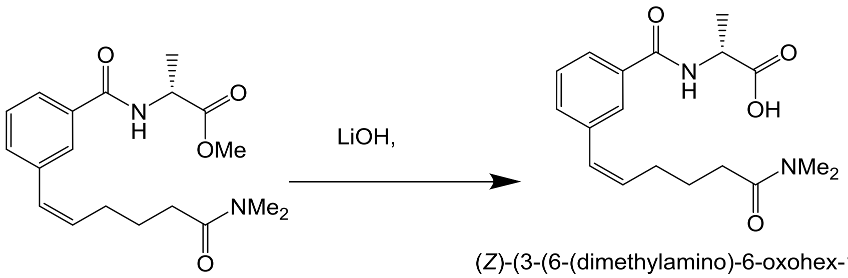

(*Z*)-(3-(6-(dimethylamino)-6-oxohex-1-en-1-yl)benzoyl)-*D*-alanine

The ester (135 mg, 0.41 mmol) in THF (2 mL) was added to lithium hydroxide, hydrate 84 mg, 2 mmol) in water (1 mL). The reaction was stirred at room temperature for 24 hrs. The THF was removed on the rotary evaporator and the residue taken up in 10% aq. Citric acid (10 mL). The aqueous mixture was extracted with DCM (3 x 30 mL) and dried over Na_2SO_4. Crude yield 307 mg. Attempts to purify using flash chromatography were unsuccessful. The product was finally purified by preparative LCMS (C18) using: Solvent A, 5% MeOH/95% H_2O, 0.1% HCOOH. Solvent B, 95% MeOH/5% H_2O, 0.1% HCOOH. Gradient 10% A to 95% over 8 min. The fractions were combined, and the volatiles removed on a rotary evaporator. The final aqueous mixture was freeze dried.

^{1}H NMR (500 MHz, $CDCl_3$) δ 9.03 (s, 1H), 7.74 (s, 1H), 7.73 – 7.67 (m, J = 7.7, 2H), 7.38 – 7.34 (m, 1H), 7.34 – 7.31 (m, 1H), 6.43 (d, J = 11.6, 1H), 5.70 (dt, J = 7.7, 11.6, 1H), 4.80 – 4.70 (m, 1H), 2.96 (s, 3H), 2.89 (s, 3H), 2.35 (t, J = 7.1, 3H), 2.32 – 2.22 (m, 1H), 1.86 – 1.73 (m, 2H), 1.54 (d, J = 7.2, 3H).

^{13}C NMR (126 MHz, $CDCl_3$) δ 175.39, 173.51, 168.12, 137.75, 133.65, 132.95, 132.31, 128.96, 128.62, 127.07, 126.06, 49.28, 37.55, 35.84, 32.64, 28.27, 25.08, 17.84.

FIGURE S1

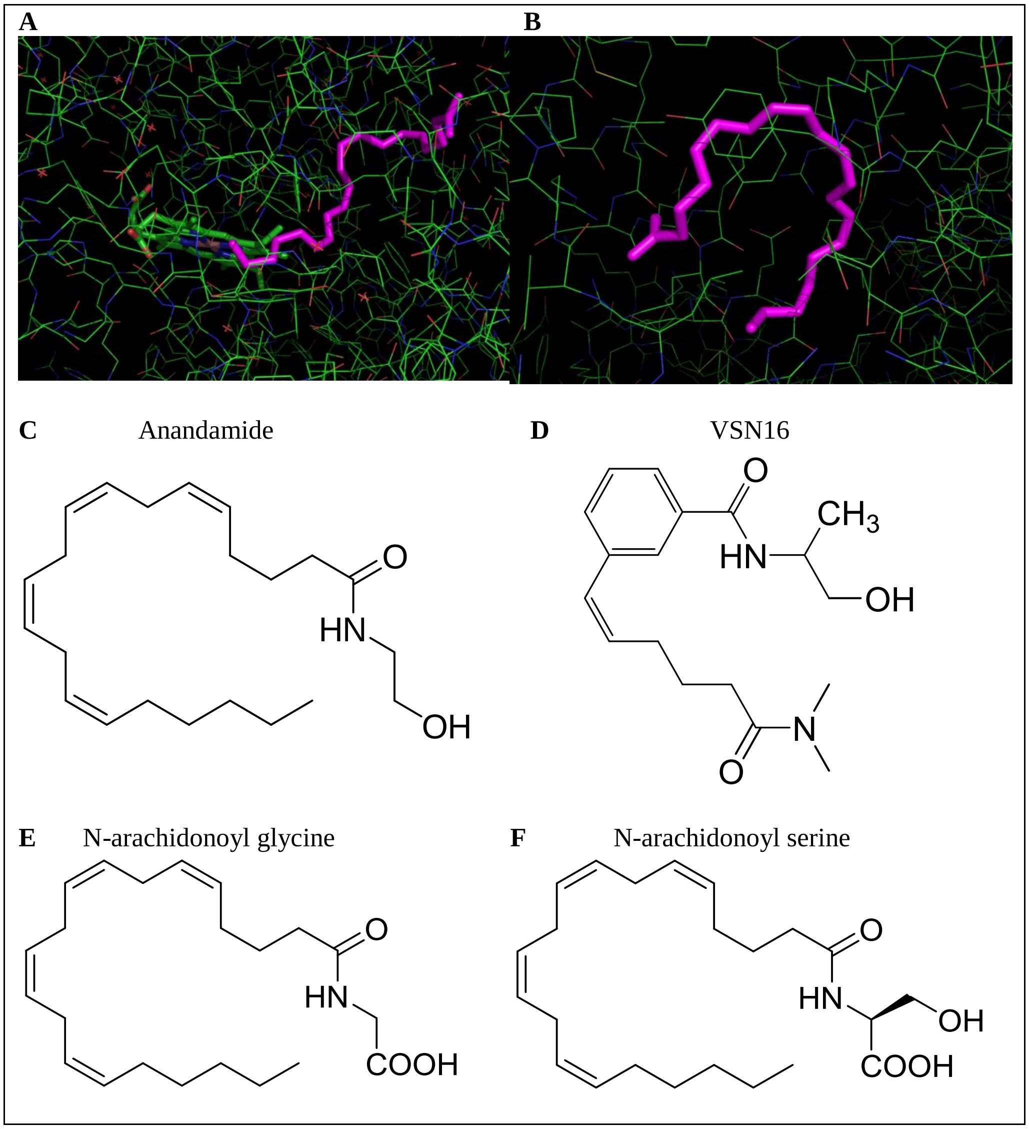

Biological conformations of the arachidonic acid chain. (**A, B**) "C" type conformations of the polyene chain from diverse biological targets from X-ray. This suggested that conformational restriction of (**C**) anandamide would be useful approach to generate novel molecules as in (**D**) VSN16. This has structural similarities with (**E**) N-arachidonoyl glycine and (**F**) N-arachidonoyl serine.

FIGURE S2.

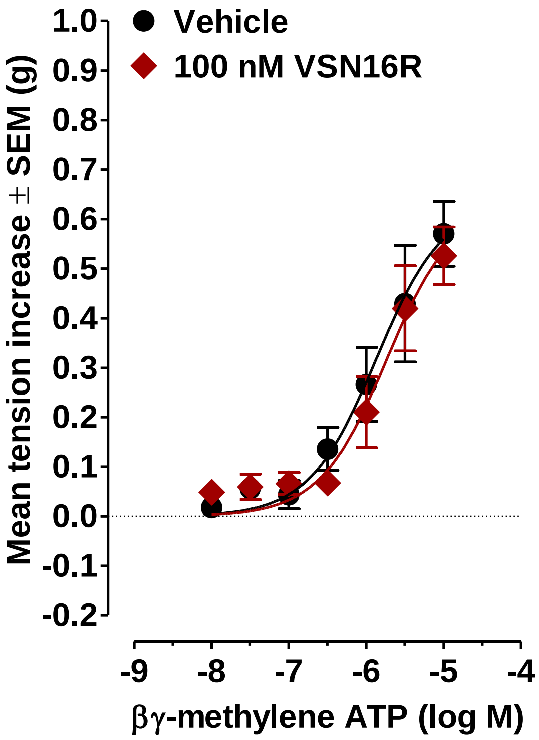

VSN16R does not inhibit beta gamma methylene adenosine triphosphatase-induced muscle contraction in the vas deferens. Mouse vas deferens were treated with either DMSO vehicle or 100 nM VSN16R 30 min before the first organ bath injection of various concentrations of βγ-methylene ATP into the organ bath . The results represent the mean ± SEM of βγ-methylene ATP-induced increases in tension (expressed in grams) of electrically unstimulated vasa deferentia. (n=6/group).

FIGURE S3

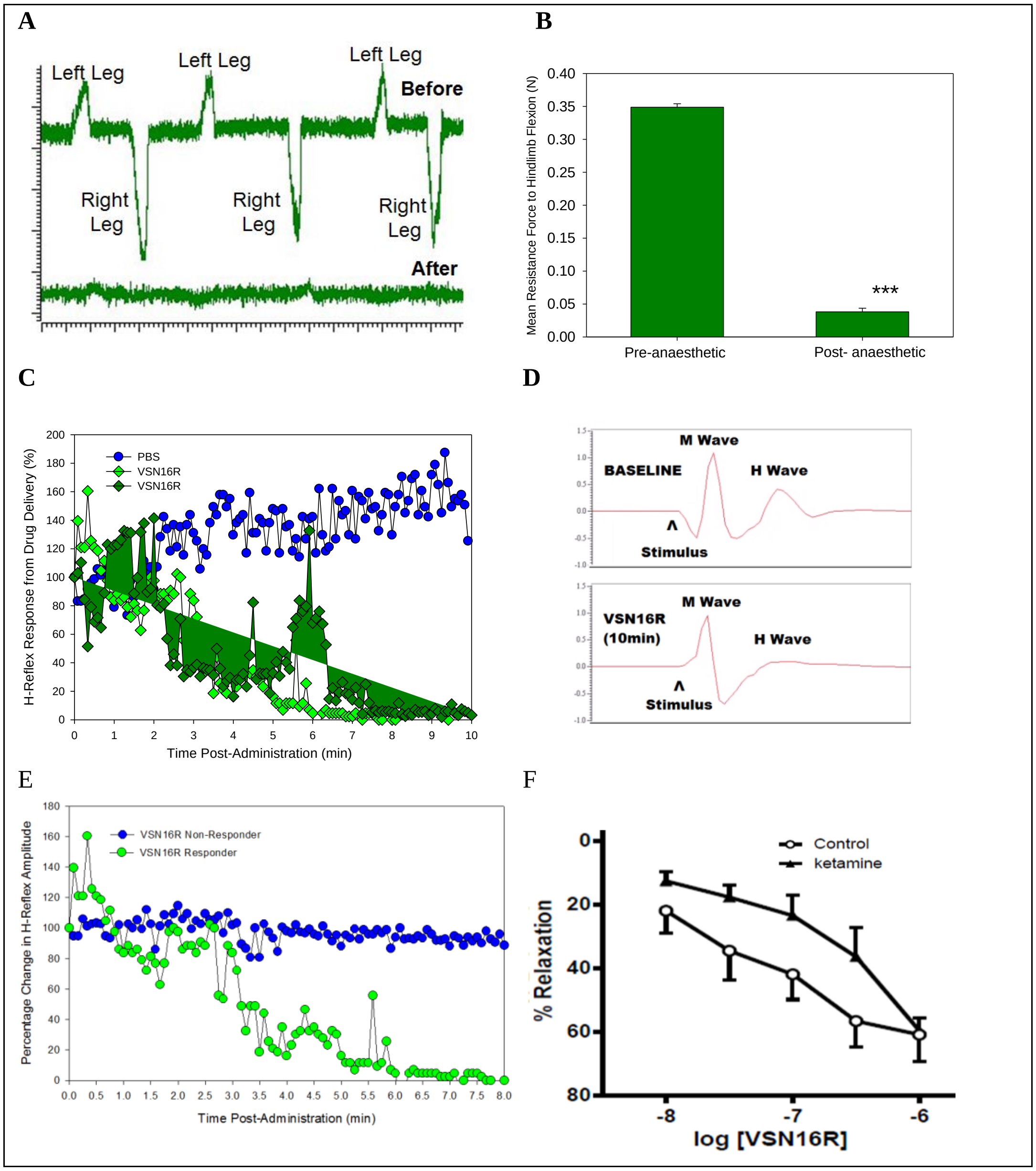

Anaesthetics inhibit spasticity and muscle tone and can interfere with the action of VSN16R.

(**A**) Strain gauge recording before and after (5min) the induction of ketamine and medetomide anesthesia, typically used for rodent electrophysiology studies. (**B**) Loss of muscle tone of spastic animals following anaesthetic showing measurement of resistance to limb flexion before and 5min after anesthesia.*** significant compared to baseline before anesthetic using paired t test (n=5 animals) (**C**) The magnitude of the H-wave was measured in the shin muscle in spastic animals following sciatic nerve stimulation (100%= H wave at administration of 30mg/kg i.v. VSN16R in phosphate buffered saline at 0min). Although n=0/3 PBS-treated animals showed an inhibition of the H reflex, this could be inhibited by VSN16R (**D**) Electrophysiological trace of the shin muscle following stimulation of a spastic mouse before and after 30mg/kg i.v. VSN16R administration. (**E**) However, the H reflex was only inhibited in some (3/5) animals responded (Green circles), whereas others did not (blue circles). Trace of individual mice (**F**) However, it was subsequently found that ketamine (200 µM. To represent anaesthetic levels in blood) can inhibit the mechanism of action of VSN16R in part of the dose-response curve to methoxamine-evoked contraction of rat mesenteric artery (n= 5-6 cultures). Ketamine blocks NMDA receptors, that limits calcium ion fluxes that can influence BK_{Ca} function and can inhibit the inside-out current of BK_{Ca} channels with an EC_{50} = ~25µM (Denson DD et al. Brain Res 638:61). In contrast to healthy animals, mice with spasticity did not tolerate anaesthetics, which caused death in some instances, prompting discontinuation of the approach prior to attempted dose reduction.

FIGURE S4

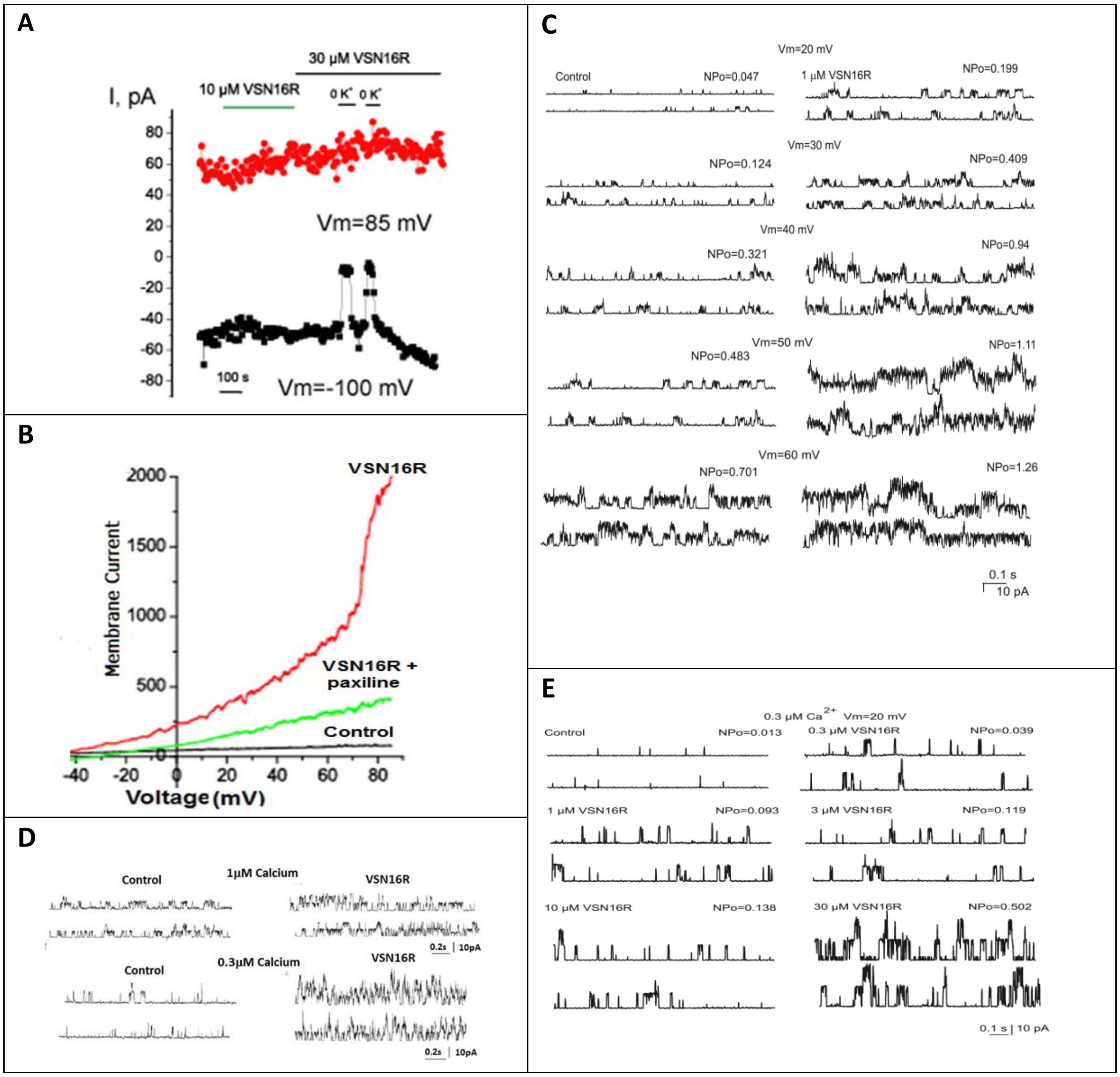

Patch Clamp analysis of VSN16R activity on BK_{Ca} channels. (**A**) Primary pig aorta do not respond to VSN16R (n=3 patches). Time course of the current development at -100 mV (lower) and +85 mV (upper) in response to VSN16R or the removal of potassium. (**B**) Whole cell currents of human EA.hy926 cells in response to voltage ramps before (control), and during exposure to 15μM VSN16R in the absence and presence of 2μM paxilline. The current represents the influence of endogenously expressed conducting ion channels within the cell, but the sensitivity to paxilline indicates that the majority response was mediated by BKca channels. (**C-E**) This was shown in single channel patch clamp experiments in EA.hy926 cells. (**C**) Voltage dependence of activity of VSN16R in inside-out patch clamp of single BK_{Ca} channels VSN16R. (**D**) Calcium dependence of activity of VSN16R. Single BK_{Ca} channel activity in inside-out patch held at +60 mV and exposed to either 1μM or 0.3μM free Ca^{2+} concentrations before (control) and after treatment with 3 μM VSN16R. (**E**) VSN16R exhibits a concentration-dependent induction of potassium currents in inside-out patch clamp of single BK_{Ca} in EA.hy926 cells. The patch was held at 20 mV and exposed to 0.3 μM free Ca^{2+} under symmetrical K^+ conditions. Representative traces that were repeated.

FIGURE S5.

Receptor binding profile of VSN16R

A *Receptors with Negligible Activity following incubation with 10μM VSN16R*

human: A_1, A_{2A}, A_3, α_1(non-selective), α_2(non-selective),β_1, AT_1, BZD, β_2, CCK_A,, CB1, CB2, D1, D2S, ET_A, GABA (non-selective), GAL2, CXCR2, CCR1, H_1, H_2, MC_4, ML_1, M_1, M_2, M_3, NK_2, NK_3, Y_1, Y_2,NT_1,δ_2, κ, μ, ORL1, 5-HT_{1A}, 5-HT_{1B}, 5-HT_{2A}, 5-HT_3, 5-HT_{5A}, 5-HT6, 5-HT_7, sst(non-selective), VIP_1, V_{1a}, Ca^{2+} channel (L verapamil site), K^+_V channel, SK^+Ca channel (KCNN2), Na^+ channel (site 2), Cl^- channel, fatty acid amide hydrolase, monoglycerol lipase, NE transporter, DA transporter, Nav 1.5 Na^+ channel, hERG Kv11.1 K^+ channel (tested to 100 μM), GPR6, GPR12, GPR23, GPR35, GPR55, GPR119, EDG1, EDG2, EDG3, EDG3, EDG4, EDG5, EDG6, EDG7, EDG8, $GABA_A$, $GABA_B$, GlyR , TrK_B, Na^+-Ca^{2+} exchanger.

B *VSN16R does not bind to Cannabinoid Receptors*

Receptor	Activity of VSN16R (Max. Tested)	Positive Control (Affinity and Assay)
CB_1 Receptor (Rat cerebellar membranes)	No Activity (300μM)	CP55,940 (IC_{50} = 0.36nM. Competitive ligand binding)
hCB_1 Receptor (CHO.CNR1)	No Activity (10μM)	CP55,940 (EC_{50} =24nM cAMP Assay)
hCB_1 Receptor (HEK293.CNR1)	No Activity (10μM)	Anandamide (IC_{50} =344nM GTPγS Binding assay)
hCB_2 Receptor (HEK293T.CNR2)	No Activity (10μM)	CP55,940 (EC_{50} =1nM cAMP assay)
hCB_2 Receptor (CHO-K1.CNR2)	No Activity (10μM)	(R)+WIN55,212 (IC_{50} =5.2nM GTPγS Binding assay)
		CP55,940 (IC_{50} =2.37nM GTPγS Binding assay)
hGPR55 (HEK293.GPR55)	No Activity (10μM)	Lysophosphoinositol (EC_{50} =1.09μM. Ca2+ Ion Flux assay)

C

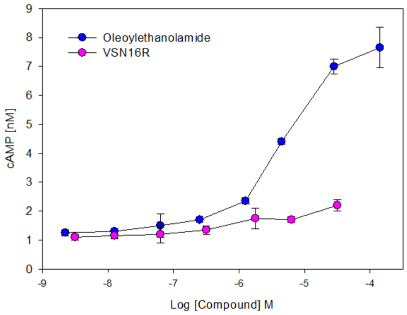

D

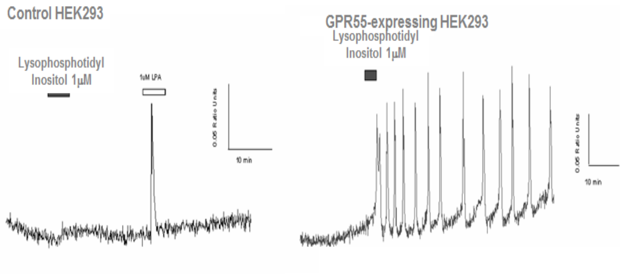

E

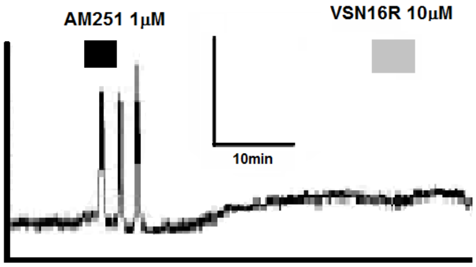

F

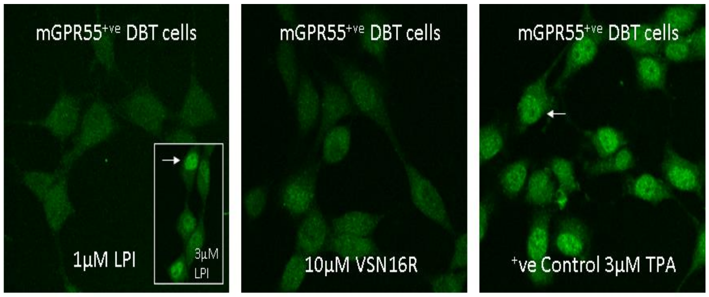

Receptors and other targets that VSN16R fails to bind/activate (**A**) Receptors lacking activity with 10μM VSN16R. Binding assays and positive controls were performed by Cerep, Multispan, DiscoverX, MDS pharma and Chantest. (**B)** Lack of activity of VSN16R on CB_1 and CB_2 cannabinoid receptors (**C**) Relative lack of activity of VSN16R agonism on U20S cells transfected with human GPR119 compared with oleoylethanolamide as assessed using cyclic AMP assay (**D**). HEK293 cells do not respond to lysophosphoinositol (LPI. GPR55 agonist) stimulation, but respond to lysophophatidic acid (LPA), unless they are transfected with human *GPR55* (left) as assessed using calcium ion fluxes (Henstridge CM et al. Br J Pharmacol 2010; 160:604). (**E**) HEK293.*GPR55* demonstrate calcium fluxes following stimulation with AM251 but do not respond to VSN16R (**F**) Lack of activity of VSN16R on DBT cells stably transfected with mouse *Gpr55*. These were incubated with 1-3μM LPI, 10μM VSN16A or the 10μM and the nuclear expression of cAMP response element-binding protein (CREB) was assessed by immunocytochemistry (Henstridge et al. 2010).

TABLE S1

VSN16R does not induce neurobehavioural behavioural tests in an Irwin test

Irwin Test Outcomes	Behavioural Effects Unchanged following VSN16R
Behavioural Profile	Alertness, Passivity, Stereotypy, Vocalizations, Transfer Reactivity, Touch, Escape, Tail-Pinch, Toe-Pinch, Pinna Reflex, Corneal Reflex, Startle Response, Visual Placing responses
Neurological profile	Body Elevation, Limb Position, Tail Elevation, Limb Tone, Grip Strength, Body Tone, Abdominal Tone, Change in Gait, Catalepsy, Righting Reflex, Twitches, Convulsion (Clonic, Tonic))
Autonomic profile	(Palpebral Size, Excretion (Urination, Diarrhoea), Secretion (Salivation, Lacrimation), Piloerection, Body Temperature change, Skin Colour (Blanch, Flush, Cyanosis) change, Respiration (Fast, Slow, Deep, Irregular) response changes or Death.

Rats were fed with 120mg/kg p.o. VSN16R in water and behavioural effects in a standard Irwin test (Roux et al. 2005) was assessed. n=5 rats.

TABLE S2

VSN16R does not induce cytochrome P450 enzymes

	IC_{50} Inhibition of substrate binding	
Enzyme	VSN16R	EC_{50} Positive Control
CYP450 1A2	>10μM VSN16R	0.28μM 4-OH-4 androstene 3,17-dione
CYP450 1A2	>10μM VSN16R	0.96μM Furafylline
CYP450 2A6	>10μM VSN16R	0.20μM Tranylcypromine
CYP450 2B6	>10μM VSN16R	2.9μM Ketoconazole
CYP450 2C19	>10μM VSN16R	8.1μM Tranylcypromine
CYP450 2C8	>10μM VSN16R	1.1μM Quercetin
CYP450 2C9	>10μM VSN16R	0.77μM Sulfaphenazole
CYP450 2D6	>30μM VSN16R	0.40μM Quinidine
CYP450 2E1	>10μM VSN16R	8.0μM 4-methylpyrazole
CYP450 3A4	>10μM VSN16R	0.08μM Ketoconazole
CYP450 3A5	>10μM VSN16R	0.36μM Ketoconazole
CYP450 3A7	>10μM VSN16R	0.45μM Ketoconazole

Inhibition of binding of either 3-cyano-7-ethoxycoumarin, dibenzylfluorescein or 7-benzyoxy-4-(trifluoromethyl) coumarin in human enzyme transfected BTI NN-5B1-4 cells or Sf9 insect cells or inhibition of dextromethorphan substrate to CYP450 2D6 in liver microsomes.

TABLE S3.
VSN16R does not induce chromosomal mutagenesis

Strain of Organism	Treatment	Dose	Revertants per plate
**First Mutation without Metabolic Stimulation**			
TA1535	DMSO	-	12.3 ± 3.1
	VSN16R	17µg	9.0 ± 6.6
		5000µg	13.7 ± 2.1
TA1537	DMSO	-	12.3 ± 4.5
	VSN16R	17µg	13.3 ± 1.5
		5000µg	7.0 ± 1.7
TA98	DMSO	-	18.7 ± 2.3
	VSN16R	17µg	15.3 ± 4.7
		5000µg	18.3 ± 7.1
TA100	DMSO	-	90.0 ± 10.6
	VSN16R	17µg	79.3 ± 7.6
		5000µg	79.3 ± 5.5
WP2uvvA	DMSO	-	4.3 ± 2.1
	VSN16R	17µg	5.3 ± 0.6
		5000µg	1.3 ± 1.2
**Second Mutation without Metabolic Stimulation**			
TA1535	DMSO	-	9.0 ± 5.61
	VSN16R	17µg	14.0 ± 5.6
		5000µg	11.3 ± 3.5
TA1537	DMSO	-	8.7 ± 3.1
	VSN16R	17µg	12.3 ± 2.9
		5000µg	10.3 ± 2.9
TA98	DMSO	-	25.7 ± 8.3
	VSN16R	17µg	23.3 ± 1.2
		5000µg	23.7 ± 1.2
TA100	DMSO	-	79.5 ± 4.9
	VSN16R	17µg	85.7 ± 6.7
		5000µg	99.3 ± 5.5
WP2uvvA	DMSO	-	6.7 ± 1.2
	VSN16R	17µg	10.3 ± 6.7
		5000µg	8.0 ± 4.6

VSN16R was incubated at various concentrations with either TA1535, TA1537, TA98 and TA100 strains of *Salmonella typhimurium*, which carry mutations in genes involved in histidine synthesis, and WP2uvvA *Escherichia coli*. The bacteria are spread on agar plates with limiting histidine content and incubated for 48 h. The number of histidine gene negative to positive revertants was assessed in two experiments. The results represent the mean ± SD. n=3/group. This was performed by Charles Rivers, Ltd, UK.

TABLE S4.
VSN16R does not induce tissue toxicology of VSN16R in rats and dogs

Tissue Analysed	Result of Tissue Analysis in Rats	Result of Tissue Analysis in Dogs
Artery, Aorta	Negative	Negative
Bone Marrow femur	Negative	Negative
Bone Marrow sternum	Negative	Negative
Bone, Sternum	Negative	Negative
Bone, Sternum	Negative	Negative
Brain	Negative	Negative
Cervix	Negative	Negative
Epididymus	Negative	Negative
Eye	Negative	Negative
Adrenal Gland	Negative	Negative
Mammary Grand	Negative	Negative
Parathyroid Gland	Negative	Negative
Pituitary Gland	Negative	Negative
Prostate Gland	Negative	Negative
Salivary Gland	Negative	Negative
Seminal Vesicle Gland	Negative	Negative
Gut associated lymphoid	Negative	Negative
tissue	Negative	Negative
Kidney	Negative	Negative
Heart	Negative	Negative
Large Intestine caecum	Negative	Negative
Large Intestine colon	Negative	Negative
Large Intestine rectum	Negative	Negative
Liver	Centrilobular hypertrophy *	Negative
Lung	Negative	Negative
Mandibular Lymph Node	Negative	Negative
Mesenteric Lymph Node	Negative	Negative
Skeletal Muscle	Negative	Negative
Nasal Cavity	Negative	Negative
Sciatic Nerve	Negative	Negative
Oesophagus	Negative	Negative
Ovary	Negative	Negative
Pancreas	Negative	Negative
Skin	Negative	Negative
Small Intestine, duodenum	Negative	Negative
Small Intestine, Ileum	Negative	Negative
Small Intestine, Jejunum	Negative	Negative
Spinal Cord	Negative	Negative
Spleen	Negative	Negative
Stomach	Negative	Negative
Testis	Negative	Negative
Thymus	Negative	Negative
Tongue	Negative	Negative
Trachea	Negative	Negative
Ureter	Negative	Negative
Urinary bladder	Negative	Negative
Uterus	Negative	Negative
Vagina	Negative	Negative

Following 28 day toxicology of feeding either: rats treated with 100mg/kg, 300mg/kg, 1000mg/kg p.o. (n-20/group) or dogs treated with 50mg/kg, 100mg/kg or 200mg/kg p.o. (n=6/group). Tissues were collected fixed in formalin, embedded in paraffin-wax sectioned and stained with hematoxylin and eosin and examined by a veterinary pathologist for evidence of toxicity. There was generally no microscopic findings attributed to treatment with VSN16R. Studies were performed by Charles Rivers, Ltd. However, * There was hypertrophy of the liver that was evident at high doses VSN16R was associated with liver weight (mean ± SD) increases: 11.0 ± 1.2g placebo, 11.6 ± 1.3g (300mg/kg p.o.), 16.7 ± 2.1g (1000mg/kg p.o. $P<0.001$ assessed using Students t test. n= 10 female rats/group) associated with liver enzyme induction, supporting enhanced VSN16R clearance in pharmacokinetic studies. This was not seen in dogs or doses below 300mg/kg in rats.

TABLE S5.
Lack of hypotension induced by VSN16R in dogs

Parameter	Time	Vehicle	50mg/kg	100mg/kg	200mg/kg
Heart Rate (bpm)	0 min	105 ± 9	104 ± 9	107 ± 9	101 ± 9
	30min	97 ± 9	104 ± 9	114 ± 9	125 ± 9
	60min	121 ± 9	119 ± 9	111 ± 9	134 ± 9
Arterial Pressure (mm Hg)	0 min	105 ± 9	102 ± 5	105 ± 5	105 ± 5
	30min	94 ± 5	97 ± 5	99 ± 5	97 ± 5
	60min	112 ± 5	106 ± 5	106 ± 5	102 ± 5
Systolic Blood Pressure (mm Hg)	0 min	136 ± 6	139 ± 6	142 ± 6	141 ± 6
	30min	130 ± 6	141 ± 6	139 ± 6	130 ± 6
	60min	151± 6	143 ± 6	138 ± 6	130 ± 6
Diastolic Blood Pressure (mm Hg)	0 min	79 ± 4	83 ± 4	86 ± 4	87 ± 1
	30min	77 ± 4	83 ± 4	86 ± 4	87 ± 1
	60min	92 ± 4	87 ± 4	91 ± 4	88 ± 4

Adult male Beagle dogs implanted previously with a DSI Physio Tel TL11-M2 D70-PCT intramuscular telemetry device received either: 50mg/kg, 100mg/kg or 200mg/kg p.o. VSN16R in water or water. The results represent the mean ± SD. n=4.

TABLE S6.
Demographics of humans in phase I double-blind, placebo-controlled trial

Dosing<br>Dose<br>Food	Single<br>25mg<br>Fasted	Single<br>50mg<br>Fasted	Single<br>100mg<br>Fasted	Single<br>200mg<br>Fasted	Single<br>200mg<br>Fed	Single<br>400mg<br>Fasted	Single<br>800mg<br>Fasted	Single<br>VSN16R<br>Total	Single<br>Placebo<br>Total
Males/Total	6/6	6/6	6/6	6/6	6/6	6/6	6/6	42/42	14/14
Age (Years)	34 ± 9	31 ± 9	30 ± 7	35 ± 8	36 ± 10	29 ± 6	36 ± 9	33 ± 8	31 ± 7
Weight (Kg)	81 ±9	81 ±9	86 ± 6	82 ± 12	82 ± 10	76 ± 6	80 ± 9	81 ± 9	81 ±13
BMI (Kg/m^2)	26 ± 3	25 ± 2	26 ± 2	25 ± 4	27 ± 3	26 ± 2	26 ± 2	26 ± 3	26 ± 3

Dosing<br>Dose<br>Food	b.i.d.<br>25mg<br>Fasted	b.i.d<br>100mg<br>Fasted	b.i.d<br>400mg<br>Fasted	b.i.d<br>VSN16R<br>Total	b.i.d<br>Placebo<br>Total
Males/Total	6/6	6/6	6/6	18/18	6/6
Age (Years)	35 ± 7	32 ± 10	31 ± 8	32 ± 8	32 ± 9
Weight (Kg)	80 ± 8	77 ± 9	68 ± 6	75 ± 9	80 ± 13
BMI (Kg/m^2)	25 ± 3	25 ± 2	22 ± 3	24 ± 3	26 ± 2

The results represent the mean ± SD. VSN16R was administered a single dose or twice daily (b.i.d.) for 7 days.

TABLE S7. *Single Dose of VSN16R did not affect haematology and blood chemistry in humans*

Outcome	Time	Placebo	25mg	800mg
**Haematology**				
Hematocrit	Day -1	0.412 ± 0.026	0.417 ± 0.015	0.423 ± 0.024
	Day 2	0.416 ± 0.021	0.425 ± 0.021	0.421 ± 0.016
Haemoglobin	Day -1	8.9 ± 0.5	9.4 ± 0.4	9.0 ± 0.6
(mmol/L)	Day 2	9.0 ± 0.5	9.4 ± 0.5	9.3 ± 0.5
Red Blood Cells	Day -1	5.01 ± 0.58	4.90 ± 0.28	4.84 ± 0.36
(10^{12} cells/L)	Day 2	5.07 ± 0.57	4.94 ± 0.30	4.87 ± 0.30
White Blood Cells	Day -1	6.2 ± 1.0	4.9 ± 1.4	5.6 ± 1.3
(10^{9} cells/L)	Day 2	5.7 ± 0.9	5.2 ± 1.2	5.0 ± 1.1
Lymphocytes	Day -1	2.1 ± 0.3	1.9 ± 1.0	1.8 ± 0.5
(10^{9} cells/L)	Day 2	1.6 ± 0.2	1.9 ± 0.6	1.8 ± 0.6
Monocytes	Day -1	0.6 ± 0.1	0.6 ± 0.2	0.5 ± 0.1
(10^{9} cells/L)	Day 2	0.6 ± 0.1	0.5 ± 0.1	0.5 ± 0.1
Neutrophils	Day -1	3.4 ± 0.7	2.4 ± 0.7	2.5 ± 1.1
(10^{9} cells/L)	Day 2	3.1 ± 0.4	2.5 ± 0.6	2.7 ± 1.2
Eosinophils	Day -1	0.2 ± 0.1	0.1 ± 0.1	0.3 ± 0.2
(10^{9} cells/L)	Day 2	0.2 ± 0.2	0.2 ± 0.1	0.3 ± 0.1
Basophils	Day -1	0.0 ± 0.0	0.0 ± 0.0	0.0 ± 0.0
(10^{9} cells/L)	Day 2	0.0 ± 0.1	0.0 ± 0.0	0.0 ± 0.0
**Serum Chemistry**				
Bilirubin	Day -1	10.8 ± 4.3	12.5 ± 5.3	9.9 ± 2.2
(µmol/L)	Day 2	14.1 ± 4.1	14.9 ± 6.1	11.4 ± 3.0
Phosphate	Day -1	1.19 ± 0.16	1.18 ± 0.18	1.30 ± 0.18
(mmol/L)	Day 2	1.13 ± 0.10	1.20 ± 0.10	1.02 ± 0.10
Potassium	Day -1	4.32 ± 0.32	4.17 ± 0.30	4.34 ± 0.28
(mmol/L)	Day 2	4.29 ± 0.26	4.27 ± 0.18	4.19 ± 0.25
Sodium	Day -1	143 ± 2	141 ± 2	143 ± 2
(mmol/L)	Day 2	142 ± 2	141 ± 2	142 ± 3
Chloride	Day -1	103 ± 2	104 ± 2	102 ± 3
(mmol/L)	Day 2	103 ± 1	104 ± 2	104 ± 1
Magnesium	Day -1	0.8 ± 0.1	0.8 ± 0.0	0.8 ± 0.1
(mmol/L)	Day 2	0.8 ± 0.1	0.8 ± 0.0	0.8 ± 0.1
Calcium	Day -1	2.39 ± 0.10	2.37 ± 0.09	2.36 ± 0.06
(mmol/L)	Day 2	2.36 ± 0.08	2.40 ± 0.08	2.35 ± 0.04
Urea	Day -1	4.8 ± 0.7	5.5 ± 1.0	5.0 ± 1.1
(mmol/L)	Day 2	4.7 ± 0.7	4.4 ± 0.9	4.7 ± 0.4
Amylase	Day -1	65 ± 17	66 ± 21	55 ± 12
(U/L)	Day 2	59 ± 15	65 ± 21	50 ± 12
Albumin	Day -1	44 ± 2	42 ± 1	42 ± 1
(g/L)	Day 2	42 ± 3	42 ± 1	42 ± 1
Globulin	Day -1	29.4 ± 2.5	28.8 ± 5.6	29.4 ± 2.2
(g/L)	Day 2	29.5 ± 3.7	28.8 ± 5.6	28.9 ± 1.2
Creatinine	Day -1	79 ± 9	85 ± 14	85 ± 14
(µmol/L)	Day 2	78 ± 11	84 ± 10	84 ± 10
Glucose	Day -1	4.55 ± 0.64	4.78 ± 0.46	4.45± 0.32
(mmol/L)	Day 2	5.06 ± 1.11	4.78 ± 0.58	5.51 ± 0.92
Triglycerides	Day -1	1.47 ± 0.68	0.85 ± 0.36	1.28 ± 0.71
(mmol/L)	Day 2	1.41 ± 0.49	1.26 ± 0.25	1.55 ± 0.61
Cholesterol	Day -1	4.57 ± 0.97	4.71 ± 0.75	4.63 ± 0.94
(mmol/L)	Day 2	4.65 ± 0.65	4.79 ± 0.77	4.68 ± 0.78
Urea	Day -1	4.8 ± 0.7	5.5 ± 1.0	5.0 ± 1.1
(mmol/L)	Day 2	4.7 ± 0.7	4.4 ± 0.9	4.7 ± 0.4

Adult males were received gelatin capsules containing placebo (n=14) or 25mg (n=6) or 800mg (n=6 humans/group) VSN16R. Plasma was collected before and after drug administration. The results represent the mean ± SD.

TABLE S8. *Single dose of VSN16R did not affect coagulation, urinanalysis, vital signs and electrocardiograms in humans*

Outcome	Time	Placebo	25mg	800mg
**Coagulation**				
Partial Prothrombin Time	Day -1	10.6 ± 0.5	10.5 ± 0.1	10.5 ± 0.4
(s)	Day 2	10.8 ± 0.5	10.8 ± 0.3	10.9 ± 0.5
Thrombin Time	Day -1	15.7 ± 0.8	16.1 ± 0.8	15.9 ± 1.2
(s)	Day 2	16.0 ± 1.0	16.2 ± 0.8	15.9 ± 1.2
Activated Partial Thrombin	Day -1	26.4 ± 1.0	26.4 ± 1.0	25.4 ± 1.5
Time (s)	Day 2	26.4 ± 0.9	26.4 ± 0.9	24.9 ± 1.9
**Urinanalysis**				
pH	Day -1	6.4 ± 0.8	6.3 ± 0.3	6.1 ± 0.8
	Day 2	6.4 ± 0.7	7.0 ± 0.6	6.0 ± 0.4
Specific Gravity	Day -1	1.018 ± 0.008	1.018 ± 0.010	1.018 ± 0.005
	Day 2	1.019 ± 0.006	1.016 ± 0.012	1.017 ± 0.009
Urine Glucose	Day -1	0.0 ± 0.0	0.0 ± 0.0	0.0 ± 0.0
(mmol/L)	Day 2	0.0 ± 0.0	0.0 ± 0.0	0.0 ± 0.0
Ketones	Day -1	0.0 ± 0.0	0.0 ± 0.0	0.0 ± 0.0
(mmol/L)	Day 2	0.0 ± 0.0	0.0 ± 0.0	0.0 ± 0.0
Urine RBC	Day -1	0.0 ± 0.1	0.0 ± 0.0	0.0 ± 0.0
(mg/L)	Day 2	0.0 ± 0.0	0.0 ± 0.0	0.0 ± 0.0
Urine Protein	Day -1	0.05± 0.04	0.10 ± 0.13	0.04 ± 0.04
(g/L)	Day 2	0.07 ± 0.09	0.08 ± 0.10	0.06 ± 0.08
Urine Bilirubin	Day -1	0.0 ± 0.0	0.0 ± 0.0	0.0 ± 0.0
(μmol/L)	Day 2	0.0 ± 0.0	0.0 ± 0.0	0.0 ± 0.0
**Vital Signs**				
Supine Respiratory Rate	Day -1	15 ± 2	14 ± 2	14 ± 2
(Breath/min)	Day 2	15 ± 2	14 ± 2	16 ± 2
Supine Temperature	Day -1	36.6 ± 0.3	36.7 ± 0.3	36.6 ± 0.2
(˚C)	Day 2	36.7 ± 0.2	36.6 ± 0.2	36.6 ± 0.2
Supine Systolic Blood	Day -1	120 ± 7	115 ± 9	117 ± 10
Pressure (mmHg)	Day 2	116 ± 9	117 ± 9	118 ± 10
Supine Diastolic Blood	Day -1	67 ± 6	67 ± 10	70 ± 10
Pressure (mmHg)	Day 2	64 ± 8	65 ± 4	71 ± 9
Supine Pulse Rate	Day -1	65 ± 13	60 ± 13	56 ± 7
(beats per min)	Day 2	66 ± 15	61 ± 15	65 ± 9
**Electrocardiograms**				
Heart Rate	Day -1	60 ± 11	58 ± 10	59 ± 7
(beats per min)	Day 1	61 ± 11	59 ± 12	67 ± 8
PR Duration	Day -1	167 ± 30	189 ± 18	172 ± 20
(msec)	Day 1	165 ± 27	184 ± 20	164 ± 16
QRS Duration	Day -1	101 ± 7	99 ± 8	97 ± 5
(msec)	Day 1	101 ± 9	98 ± 9	98 ± 7
RR Duration	Day -1	1026 ± 228	1061 ± 193	1030 ± 107
(msec)	Day 1	1013 ± 149	1044 ± 185	903 ± 122
QT Duration	Day -1	393 ± 29	399 ± 26	403 ± 21
(msec)	Day 1	391 ± 25	388 ± 26	383 ± 19

Adult males were received gelatin capsules containing placebo (n=14) or 25mg (n=6) or 800mg (n=6 humans/group) VSN16R. The results represent the mean ± SD. Plasma samples were taken before and after dosing. 12-lead electrocardiograms using a Mortara machine and each lead was recorded for at least 3 beats at a speed of 25 mm/s and recorded.

TABLE S9. *Lack of hypotension induced by VSN16R in humans*

Parameter	Time	Placebo	25mg	800mg
Heart Rate (bpm)	0 min	57 ± 12	54 ± 9	63 ± 12
Supine	30min	59 ± 10	55 ± 10	57 ± 6
	60min	57 ± 10	54 ± 9	59 ± 6
Systolic Blood Pressure (mm Hg)	0 min	115 ± 8	116 ± 8	115 ± 9
Supine	30min	114 ± 6	115 ± 7	115 ±9
	60min	114 ± 8	114 ± 5	117 ± 7
Diastolic Blood Pressure (mm Hg)	0 min	66 ± 8	66 ± 7	72 ± 13
Supine	30min	66 ± 7	65 ± 5	68 ± 14
	60min	64 ± 6	63 ± 6	72 ± 8
Heart Rate (bpm)	0 min	79 ± 15	73 ± 9	76 ± 22
Standing	30min	75 ± 12	72 ± 8	81 ± 10
	60min	80 ± 11	75 ± 9	84 ± 11
Systolic Blood Pressure (mm Hg)	0 min	121 ± 9	121 ± 16	123 ± 8
Standing	30min	118 ± 10	120 ± 6	120 ± 6
	60min	117 ± 9	119 ± 8	120 ± 8
Diastolic Blood Pressure (mm Hg)	0 min	78 ± 6	74 ± 6	76± 10
Standing	30min	72 ± 11	75 ± 6	80 ± 9
	60min	73 ± 7	75 ± 7	81 ± 10

Adult males were received gelatin capsules containing placebo (n=14) or 25mg (n=6) or 800mg (n=6) VSN16R. The results represent the mean ± SD. Pulse rate and blood pressure was measured after laying down for 10 min or standing.
